# Comparison of the effectiveness of high-intensity laser therapy versus low-level laser therapy in musculoskeletal disorders: a systematic review and network meta-analysis

**DOI:** 10.1007/s10103-026-04812-9

**Published:** 2026-02-16

**Authors:** Hernán Andrés de la Barra Ortiz, Nivaldo Antonio Parizotto, Richard Eloin Liebano

**Affiliations:** 1 Exercise and Rehabilitation Sciences Institute, School of Physical Therapy, Faculty of Rehabilitation Sciences, Universidad Andres Bello, Santiago de Chile, 7591538, Chile, Andrés Bello National University, Santiago, Chile; 2 Physiotherapeutic Resources Research Laboratory, Department of Physical Therapy, Federal University of São Carlos, São Carlos, Brazil; 3 Physical Therapy Department, Federal University of São Carlos, São Carlos, Brazil; 4 Biomedical Engineering, São Paulo, Brazil, Universidade Brasil, São Paulo, Brazil; 5 Department of Rehabilitation Sciences, University of Hartford, West Hartford, United States

**Keywords:** Musculoskeletal pain, Musculoskeletal diseases, Pain management, Laser therapy, Low-Level light therapy, High-Intensity laser therapy

## Abstract

Musculoskeletal disorders (MSDs) are a leading cause of global disability. Low-Level Laser Therapy (LLLT) and High-Intensity Laser Therapy (HILT) are widely used for pain relief, yet their comparative effectiveness remains unclear, warranting further investigation. This systematic review aims to compare the effectiveness of HILT and LLLT in the treatment of MSDs. A systematic search for randomized controlled trials (RCTs) comparing HILT and LLLT in MSDs was conducted across Web of Science, ScienceDirect, PubMed, Scopus, EBSCOhost, Cochrane Library, the Physiotherapy Evidence Database (PEDro), and Google Scholar (last updated on December 13, 2025). Pain intensity was the primary outcome, whereas disability and range of motion (ROM) were secondary outcomes. A meta-analysis was conducted using mean differences (MDs) for pain intensity outcomes (Visual Analogue Scale [VAS], Numeric Pain Rating Scale [NPRS], and the KOOS pain subscale) and standardized mean differences (SMDs) for disability and range of motion (ROM) to compare the effects of HILT and LLLT. A network meta-analysis was subsequently performed to compare and rank the relative effectiveness of all laser-based treatment modalities, whether applied alone or in combination with other therapeutic interventions. Study quality was assessed using the Cochrane Risk of Bias 2 tool, and the certainty of the evidence was evaluated according to the GRADE approach. Twenty-two RCTs (n = 1,353) were included, with 575 participants receiving HILT and 778 receiving control interventions, including LLLT (n = 526), placebo laser (n = 216), and active non-laser treatments (n = 82). The overall risk-of-bias assessment indicated that 54.5% of the included studies presented methodological issues. Statistically significant reductions in pain intensity at the end of treatment favored HILT over LLLT in some comparisons, including HILT applied alone (MD = 0.8; 95% CI 0.5–1.2), HILT combined with exercise (MD = 0.6; 95% CI 0.04–1.1), and HILT combined with other physiotherapeutic interventions compared with corresponding LLLT-based approaches (MD = 1.4; 95% CI 0.6–2.2). However, the magnitude of pain reduction was generally modest and frequently below established minimal clinically important difference (MCID) thresholds. Evidence regarding disability and ROM outcomes was limited and heterogeneous, precluding firm conclusions and showing no clear statistically or clinically meaningful differences between interventions. Network meta-analysis ranked mild-dose HILT combined with exercise among the highest for pain reduction (SUCRA = 79.3%); however, ranking estimates were characterized by substantial uncertainty. According to GRADE, the clinical importance of the observed effects across pain, disability, and ROM outcomes ranged from not important to moderate, while the certainty of evidence was predominantly very low due to heterogeneity, imprecision, indirectness, and suspected publication bias. HILT demonstrated statistically greater pain reduction than LLLT in some comparisons, particularly when combined with exercise or other physical therapy modalities. However, these effects were generally modest and supported by very low certainty of evidence. Evidence regarding disability and ROM outcomes remains insufficient, particularly in terms of clinical relevance. Based on the current evidence, both HILT and LLLT may be considered therapeutic options for pain management in MSDs; however, treatment decisions should be made cautiously within a multimodal rehabilitation framework. Furthermore, adherence to WALT dosage recommendations for LLLT is advised to enhance treatment consistency and optimize clinical outcomes.

**Supplementary Information:**

The online version contains supplementary material available at 10.1007/s10103-026-04812-9.

## Introduction

Musculoskeletal disorders (MSDs) impact more than 1.7 billion individuals worldwide, leading to substantial disability and healthcare costs [1, 2]. Common conditions like low back pain, neck pain, and osteoarthritis lead to high socioeconomic burdens through increased healthcare expenses, absenteeism, and decreased productivity. Many MSDs, particularly degenerative ones like osteoarthritis, tend to progress to chronicity, resulting in persistent pain and functional impairment [1, 3]. Chronic musculoskeletal pain, lasting over three months, affects 20–33% of the population and remains a leading cause of disability [3, 4].

The incidence of MSDs is rising, primarily due to aging populations, sedentary lifestyles, and occupational risk factors [1, 2]. Chronic pain is often linked with psychological distress, contributing to increased anxiety and depression [3]. The pathophysiology of MSDs involves complex interactions between the musculoskeletal and nervous systems, leading to central sensitization and altered pain processing. These factors complicate management, highlighting the need for comprehensive, multidisciplinary approaches for long-term care and prevention [5].

Laser therapy has emerged as a key non-invasive intervention for MSDs, leveraging photobiomodulation to alleviate pain and promote tissue repair [6–8]. This process entails the absorption of laser light by biological photoacceptors, including cytochrome c oxidase, water molecules, and chromophores in the cell membrane, triggering mitochondrial activation, adenosine triphosphate (ATP] synthesis, gene expression, and modulation of the inflammatory process [6, 9].

Low-level laser therapy (LLLT), with an power output under 0.5 W (500 mW), is commonly used in physical therapy to manage MSDs, mainly for its effects on reducing inflammation, alleviating pain, and promoting tissue healing [6, 8]. The mechanisms underlying LLLT are primarily based on photobiomodulation and include activation of the electron transport chain, improved microcirculation, reduction of oxidative stress, enhanced endorphin release, decreased nociceptive nerve conduction, and modulation of inflammatory processes. Collectively, these effects contribute to pain relief, tissue repair, and the control of inflammation and edema [7, 8]. Recently, High-Intensity Laser Therapy (HILT) has been introduced as a newer approach in rehabilitation, using power levels above 0.5 W and longer wavelengths within the infrared spectrum (900–1,064 nm) [9–12]. HILT has emerged as an advanced laser therapy technology for managing musculoskeletal pain, claiming potential advantages over LLLT [10–13]. Although both modalities share the same fundamental mechanism—photobiomodulation—HILT delivers higher energy doses in a shorter period due to its greater power output and can induce thermal effects that may further enhance tissue response and therapeutic efficiency [11–14].

Despite these theoretical advantages, HILT remains a substantially more expensive technology—often several times the cost of LLLT devices—which may limit its accessibility and sustain the continued clinical preference for LLLT. Previous systematic reviews (SR) up to 2022 included a limited number of trials and yielded inconclusive findings, lacking direct comparisons or advanced synthesis methods such as network meta-analysis [14]. Moreover, several new randomized controlled trials (RCTs) published after 2022 may refine the current understanding of the relative effectiveness of these modalities.

Considering the growing clinical interest in HILT for the management of musculoskeletal disorders and its higher technological cost compared with LLLT, it is essential to determine whether its purported advantages translate into superior therapeutic outcomes. Therefore, this SR and network meta-analysis aimed to compare the effectiveness of HILT and LLLT in the management of MSDs, providing updated evidence to support clinical decision-making, optimize healthcare resource allocation in rehabilitation practice, and guide future research on photobiomodulation-based therapies.

## Methods

### Study design

Non-experimental quantitative SR with secondary analysis, reported in adherence to Preferred Reporting Items for SR and Meta-Analyses (PRISMA) guidelines and registered in the International Prospective Register of SR (PROSPERO) (CRD42025630607; 22 December 2024) [15].

### Selection criteria

This SR follows the PICOS (Population, Intervention, Comparison, Outcomes, and Study type] framework [17], evaluating patients with MSDs treated with HILT, alone or in combination with other interventions, compared to LLLT, either alone or with additional treatments. The primary outcome was pain intensity, assessed using scales such as the Visual Analog Scale (VAS), the Numeric Pain Rating Scale (NPRS), or others. Secondary outcomes included disability—measured with tools such as the Knee Injury and Osteoarthritis Outcome Score (KOOS), the Western Ontario and McMaster Universities Osteoarthritis Index (WOMAC), the Oswestry Disability Index (ODI), and the Disabilities of the Arm, Shoulder, and Hand (DASH)—as well as range of motion (ROM), when reported. All outcomes were assessed pre- and post-treatment. Studies using multiple instruments for the same outcome were analyzed qualitatively, prioritizing the most reliable measures. Only randomized controlled trials (RCTs) were included, with no language restrictions. Exclusion criteria included musculoskeletal disorders with neurological involvement, interventions targeting non-MSD conditions, absence of a direct HILT–LLLT comparison, inaccessible or incomplete data, and lack of relevant outcomes for this review. Complementary laser applications such as laser acupuncture and auriculotherapy were also excluded, as these techniques stimulate acupuncture points or auricular regions to induce systemic neuromodulation rather than direct photobiomodulation of musculoskeletal tissues [16].

### Database search

An electronic search for RCTs comparing HILT versus LLLT in MSDs was performed across PubMed, Web of Science, Scopus, EBSCOhost, ScienceDirect, and the Cochrane Library, with an additional manual search in the Physiotherapy Evidence Database (PEDro) and Google Scholar [18]. The search strategy included the keywords *“Lasers*,*” “Laser Therapy*,*” “Low-Level Light Therapy*,*” “High-Intensity Laser Therapy*,*” “Class IV Laser*,*” “Musculoskeletal Pain*,*” “Musculoskeletal Diseases*,*” “Neck Pain*,*” “Myofascial Pain Syndromes*,*” “Low Back Pain*,*” “Osteoarthritis”*,* and “Pain Management”.* Results were retrieved for individual terms and their combinations using the Boolean operators *“OR” and “AND.”* No filters were applied to maximize article retrieval from each database.

### Data extraction

The selection of RCTs was performed using the Rayyan web app [19], which enabled independent screening and duplicate removal throughout the selection process. Three reviewers (HDB, NAP, and REL) independently assessed all potentially eligible studies, resolving any discrepancies through discussion and consensus within the research team. Data extraction was subsequently conducted using a structured form developed in Microsoft Excel. Extracted information included bibliographic details (authors, year of publication, and journal), participant characteristics (sample size, age, and sex), and clinical information related to the musculoskeletal condition investigated, including pain intensity at baseline and after the intervention. Secondary outcomes—such as disability, range of motion, and other functional measures—were also collected when available. Detailed laser parameters were recorded for both HILT and LLLT, including wavelength, power, energy dose, session duration, and total number of sessions. When essential or unclear data were identified, the corresponding author of the original trial was planned to be contacted by email for clarification. The methodological quality of each RCT was initially verified in the PEDro database. For studies not indexed, quality assessment was independently conducted by the review team using the PEDro scale through consensus-based evaluation.

### Risk of bias in RCTs

The risk of bias (RoB) of the included randomized controlled trials was assessed using the Cochrane Risk of Bias 2 (RoB 2) tool [20], which evaluates five domains: (i) bias arising from the randomization process, (ii) deviations from intended interventions, (iii) missing outcome data, (iv) bias in outcome measurement, and (v) selective reporting. Each trial was classified as having a low risk, some concerns, or a high RoB. A study was judged to have an overall high RoB if at least one domain was rated as high risk. Studies with two or more domains rated as having some concerns were also classified as having an overall high RoB, in accordance with the RoB 2 algorithm [20]. Inter-rater agreement among reviewers (HDB, NAP, and REL) was calculated using Cohen’s kappa statistic [20]. The Robvis tool was used to generate traffic-light and summary plots to visualize the distribution of risk-of-bias judgments across studies (https://www.riskofbias.info/welcome/robvis-visualization-tool) [21].

### Statistical analysis

A meta-analysis was conducted to perform direct comparisons of the main outcomes when data from at least two studies were available. Statistical analyses were performed using Review Manager (RevMan, version 5.4). Pooled mean differences (MDs) were calculated for continuous outcomes assessed with equivalent or comparable scales (e.g., VAS, NPRS, KOOS pain subscale), whereas standardized mean differences (SMDs) were used when different instruments measured the same construct. When a study reported the same outcome using more than one measurement instrument, data from the instrument most frequently reported across the included studies (e.g., VAS) were selected to ensure methodological consistency and comparability. When outcomes were reported as medians and interquartile ranges (IQRs; 25th percentile [Q1] and 75th percentile [Q3]), these values were converted into means and standard deviations using the formulas proposed by Wan et al. (2014) to allow their inclusion in the quantitative synthesis [22]. The mean was estimated as Mean = (Q1 + Median + Q3)/3, and the standard deviation was calculated using SD = (Q3 − Q1)/{2 × Φ⁻¹ [(0.75 × *n* − 0.125)/(*n* + 0.25)]} [22].

For each study, changes in pain intensity, disability, and ROM were calculated as the difference between baseline and post-treatment values (T0-T1), and between baseline and follow-up values (T0-T2) when available. When multiple follow-ups were available, the first was analyzed to ensure consistency across studies. When the standard deviation of change (SD change) between baseline and post-treatment values was not reported, it was estimated using the Cochrane formula: SD change = √(SD_pre² + SD post² − 2 × r × SD pre × SD post). A correlation coefficient (r) of 0.7 between baseline and post-treatment measures was assumed, as recommended by the Cochrane Handbook for SRs of Interventions [23], providing a balanced estimation between conservative and overestimated variance assumptions. Pooled effect estimates with 95% confidence intervals (CIs) were obtained using the inverse variance method, which assigns greater weight to studies with smaller variance [24]. The meta-analysis compared the effects of HILT and LLLT, either applied as standalone interventions or combined with other therapeutic modalities. In three-arm studies, split comparisons were performed in accordance with Cochrane recommendations [24], prioritizing concurrent analyses of HILT versus LLLT and their respective combinations with additional interventions. Heterogeneity was assessed using the chi-squared test and the I² statistic (α = 0.05), with substantial heterogeneity defined as I² ≥ 50% [25]. Depending on the level of heterogeneity, either the DerSimonian–Laird random-effects model or the Mantel–Haenszel fixed-effects model was applied [26]. SMDs were interpreted according to Cohen’s d, categorized as small (0.2–0.5), moderate (0.5–0.8), or large (≥ 0.8) effects [27, 28]. For outcomes expressed as MDs, the magnitude of the effect was interpreted relative to the scale of measurement, with changes < 10% considered small, 10–20% considered moderate, and > 20% considered large. These thresholds were used to aid interpretation of clinical relevance and are consistent with methodological approaches commonly applied in systematic reviews, including Cochrane reviews [28].

Additionally, a network meta-analysis was conducted to rank the comparative probability of efficacy among different treatment modalities involving HILT and LLLT, applied either as standalone interventions or combined with other physical therapy approaches, allowing indirect comparisons across the included RCTs [29]. Nodes represented individual treatments, with node size proportional to the number of participants and line thickness indicating the number of RCTs comparing those interventions. Model transitivity was considered acceptable when qualitative comparisons of clinical and methodological characteristics across RCTs indicated sufficient similarity between interventions, while model fit was evaluated using the mean deviance (D̄) [29]. Heterogeneity was assessed using the τ statistic, and inconsistency was examined through the node-splitting method with a significance level of α = 0.05. Treatment efficacy was ranked using the surface under the cumulative ranking curve (SUCRA), where higher values indicate a greater probability of effectiveness. Results were displayed as Litmus Rank-O-Gram plots [30].

Publication bias was assessed for outcomes that showed statistically significant pooled effects in the meta-analysis. When at least ten studies were available for a given comparison, asymmetry was explored through visual inspection of funnel plots and statistically tested using Egger’s regression test [31].

### Clinical relevance

Clinical relevance was interpreted using predefined thresholds of effect magnitude. Effects were considered small when the mean difference was < 10% of the scale (e.g., < 10 mm on a 100-mm VAS) or when the SMD was ≤ 0.5; moderate when the MD ranged from 10% to 20% or the SMD was between 0.5 and 0.8; and large when the MD exceeded 20% or the SMD was ≥ 0.8 [27, 28]. These thresholds have been commonly applied in systematic reviews as pragmatic criteria to aid interpretation of effect sizes when condition-specific MCIDs are unavailable [29]. From a clinical perspective, a change of approximately 1.26 (95%CI 0.9 to 1.5) points on the 0–10 VAS—equivalent to a 13% improvement—has been established as the MCID [32].

### Quality of evidence

The quality of evidence regarding the effectiveness of HILT versus LLLT for MSDs was assessed using the Grading of Recommendations, Assessment, Development, and Evaluation (GRADE) approach, considering RoB, inconsistency, indirectness, imprecision, and publication bias [33]. RoB was considered when methodological limitations identified using the RoB 2 tool were deemed likely to influence effect estimates. Inconsistency was assessed based on statistical heterogeneity in the meta-analyses, with substantial inconsistency considered when heterogeneity (I^2^) exceeded 50%. Indirectness was considered when pooled analyses combined different musculoskeletal conditions and variable intervention durations, which may limit the direct applicability of the estimated effects to specific clinical scenarios. Imprecision was judged based on the width and clinical interpretation of the confidence intervals; according to GRADE guidance, imprecision was considered present when the 95% confidence interval included values that would lead to different clinical decisions. Depending on the effect measure, this corresponded to confidence intervals crossing the MCIDs for outcomes analyzed using mean differences or crossing the line of no effect or relevant effect size thresholds for outcomes analyzed using SMDs. Publication bias was assessed using funnel plots and Egger’s regression test when sufficient studies were available; for outcomes reported in fewer than three studies, publication bias could not be formally assessed. The certainty of evidence was rated as high, moderate, low, or very low. Outcome importance (not important, important, or critical) was defined a priori according to GRADE guidance, based on relevance for clinical decision-making [33, 34]. For this review, pain intensity and disability were classified as critical outcomes, given their direct relevance to patient-centered benefit and clinical decision-making. In contrast, ROM was classified as an important outcome, as it reflects functional change with a more indirect impact on patient-perceived benefit [33, 34].A Summary of Findings table was generated using GRADEpro GDT, developed by McMaster University (https://gdt.gradepro.org/app/).

## Results

### Database search

**Fig. 1:**
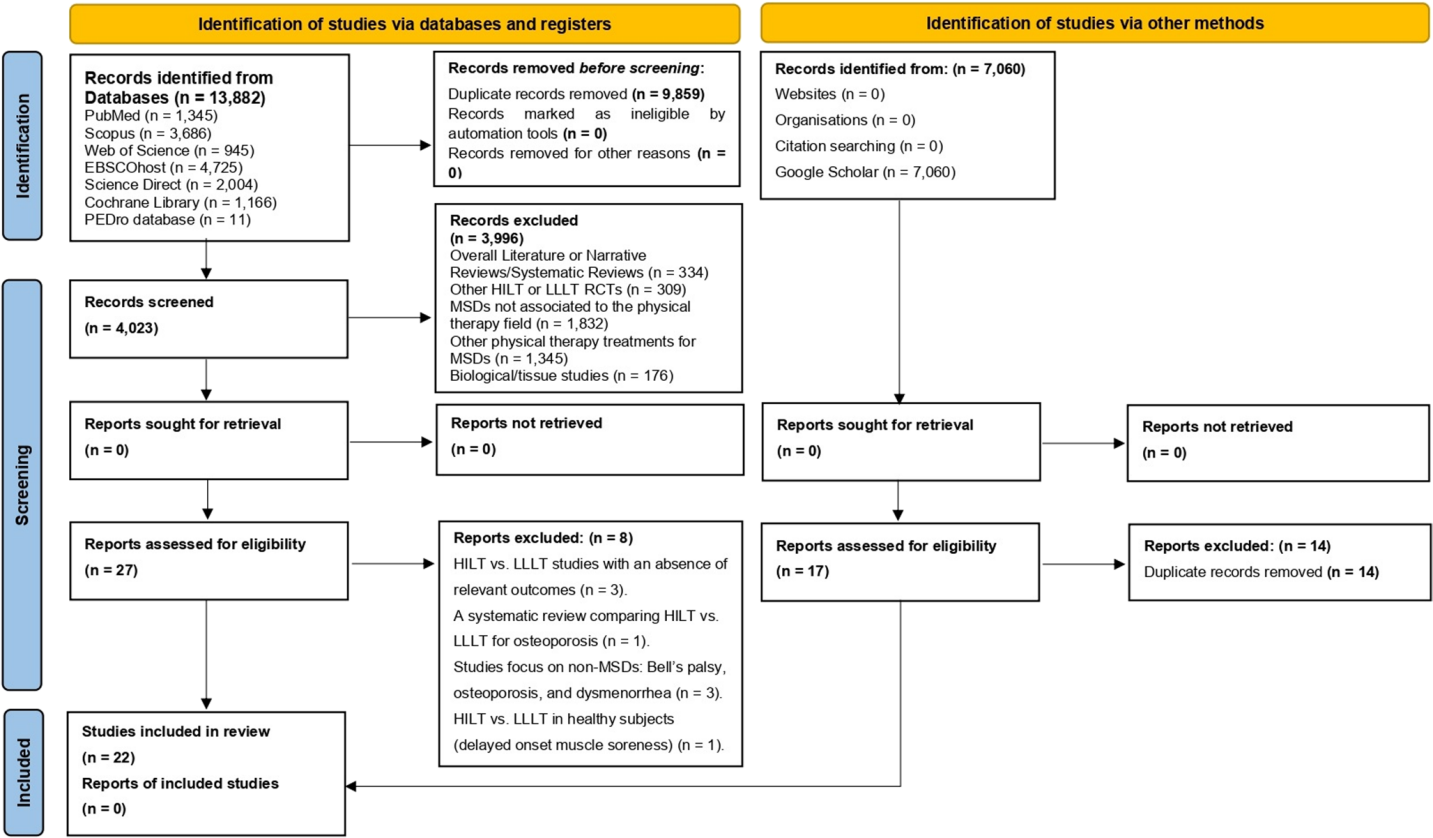
PRISMA flow chart

Results The systematic search retrieved 13,882 records (last updated on December 13, 2025). Alternative search methods identified an additional 7,060 studies. After removing duplicates, 4,023 records remained. Following title and abstract screening, 27 studies were deemed potentially eligible. Eight trials comparing HILT and LLLT were excluded due to the absence of relevant outcomes (*n* = 3), being a SR (*n* = 1), focusing on non-musculoskeletal disorders (*n* = 3; Bell’s palsy, osteoporosis, and dysmenorrhea), or investigating delayed-onset muscle soreness in healthy subjects (*n* = 1). The Google Scholar search identified 17 documents, 14 of which were duplicates, yielding three additional eligible studies. In total, 22 articles were included [35–56]. Figure 1 shows the PRISMA flow diagram summarizing the selection process [15]. Appendix 1 details the complete search strategy and results, whereas Appendix 2 lists the excluded studies.

### Risk of bias assessment

**Fig. 2:**
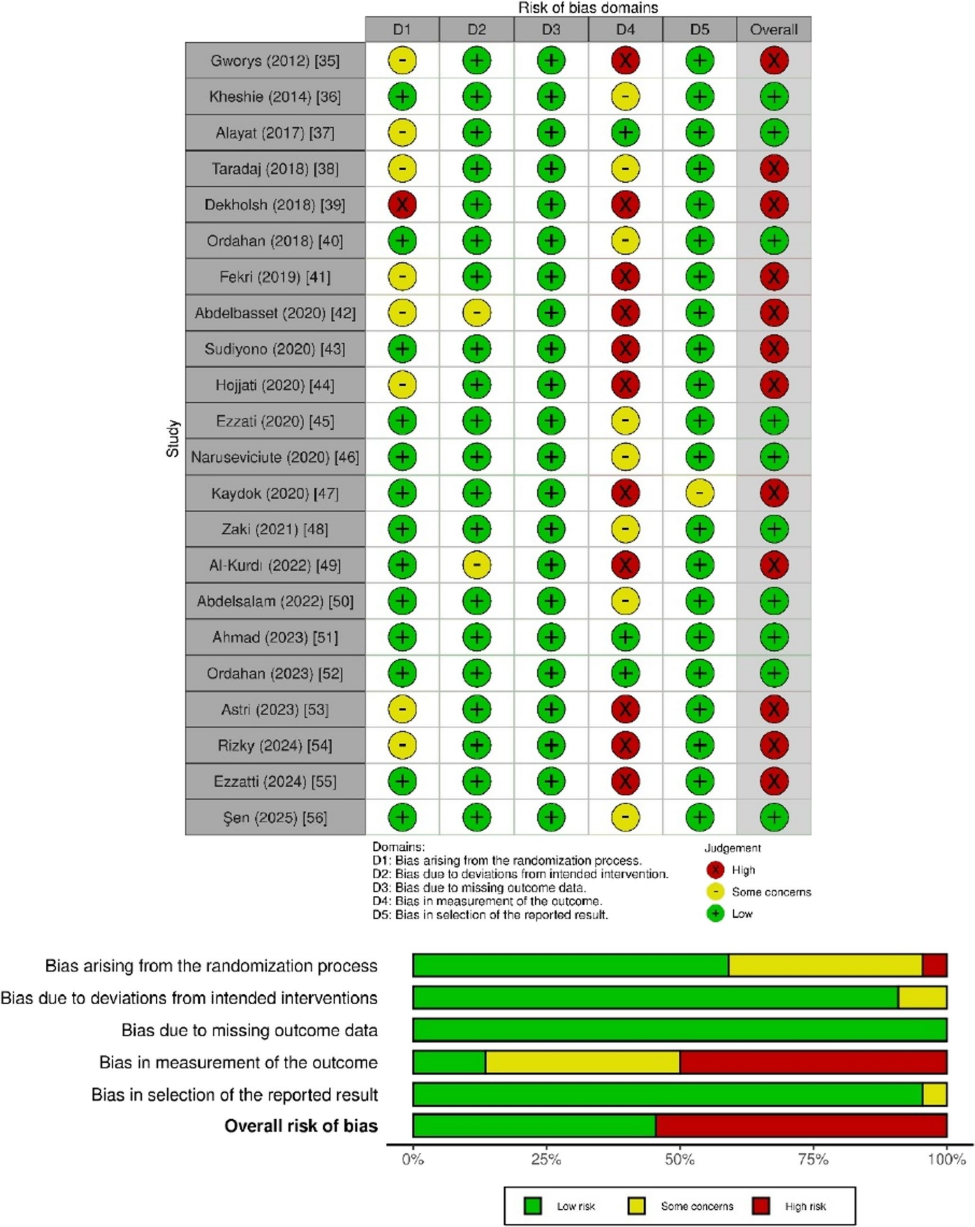
Overview of Risk of Bias Assessment

Figure 2 presents the RoB assessment, showing substantial inter-rater agreement among reviewers (κ = 0.89) [21]. The main sources of bias were related to outcome measurement (86%) and, to a lesser extent, to the randomization process (40.9%). In contrast, a lower RoB was observed for missing outcome data (100%), selection of reported results (95.5%), and deviations from intended interventions (95.2%). The overall RoB was rated as 54.5% [20]. Additionally, study quality was revised using the PEDro scale, revealing lower adherence to criteria 6, 7, and 9, which reflect essential aspects of blinding procedures and intention-to-treat analysis (Appendix 3)

### Studies characteristics

Table 1 summarizes the studies, providing details on countries, study groups, participant selection criteria, interventions, assessments, and key outcomes. The studies were conducted in Poland [32, 35], Saudi Arabia [36, 42], Iran [39, 41, 44, 45, 48], Turkey [40, 47, 49, 52, 56], Egypt [37, 50], Indonesia [43, 53, 54], Lithuania [46], and Malaysia [51], covering the period from 2012 to 2024. The MSDs studied were knee osteoarthritis [35, 37, 40, 52, 54–57] carpal tunnel syndrome [43–45], lumbar disc herniation [38, 49, 50] plantar fasciitis [40, 46], lateral epicondylitis [41, 47], chronic neck pain [37], non-specific low back pain [42], subacromial impingement syndrome [48], and frozen shoulder [52]. The studies included a total of 1,352 patients, comprising 461 males and 606 females, with an average age of 49.7 (SD = 7.5) years. Sex was unspecified in three studies [35, 41, 55]. A total of 575 participants were allocated to the experimental group (EG) and 824 to the control group (CG). Placebo interventions were used in 7 studies, involving 160 participants. Twenty-one participants dropped out across three RCTs [36, 40, 55]. In the EG, 145 participants received HILT alone, while 430 received it combined with additional interventions, including exercise [36, 37, 42, 45, 49, 50, 52, 53, 55, 56] and physical therapy such as transcutaneous electrical nerve stimulation (TENS) [39, 41, 55], therapeutic ultrasound (US) [39, 41], cryotherapy [41, 46], hot packs [39], manual therapy [51], splints [44], stretching [40, 46, 55], insoles [40, 46], kinesiology tape [48], or elbow bandages [47]. In the CGs, 71 participants received LLLT alone, while 455 received LLLT combined with other physical therapy interventions comparable to those used in the EGs. Additionally, 216 participants were allocated to placebo laser groups, and 82 to active non-laser control interventions, such as therapeutic exercise or other physical therapy modalities. The treatment frequency ranged from 1 to 5 sessions per week (average: 2–3 sessions), and the total treatment duration varied between 2 and 12 weeks.

**Table 1:** Study characteristics comparing HILT versus LLLT for MSDs

First author (year) Country	MSDs	PEDro score	Participants (*n*) mean age (SD)	Groups (*n*)	Sessions	Outcomes (instrument)	Assessment instances	Results after treatment	Conclusion	Adverse effects	Source of funding
Gworys (2012) [35] Poland	Knee OA	5/10	125 (M NS, F NS) 64.1 (10.4) years Dropout 0	EG1 (30): HILT EG2 (30): HILT mild dose CG (34): LLLT PG (31): HILT placebo	5s/week for 2 weeks	(A) PI (VAS) (B) PI (Lattinen index) (C) Disability (Lequesne index)	T0: baseline T1: post-treatment (2 weeks)	EG1: ↓PI* and ↓Disability* EG2: ↓PI* and ↓Disability* CG: ↓PI* and ↓Disability* PG: ↓PI and ↓Disability	LLLT, high-dose HILT, and mild-dose HILT improved knee function and reduced pain in OA, with high-dose HILT showing the greatest effect.	None	NS
Kheshie (2014) [36] Saudi Arabia	Knee OA	7/10	53 (M 53, F 0) 54.6 (8.5) years Dropout 8	EG (20): HILT + EX CG (18): LLLT + EX PG (15): HILT placebo + EX	2s/week for 6 weeks	(A) PI (VAS) (B) PI (WOMAC) (C) Stiffness (WOMAC) (D) Disability (WOMAC)	T0: baseline T1: post-treatment (6 weeks)	EG: ↓PI* (VAS), ↓PI* (WOMAC), ↓Stiffness*, and ↓Disability* CG: ↓PI* (VAS), ↓PI* (WOMAC), ↓Stiffness*, and ↓Disability* PG: ↓PI* (VAS), ↓PI* (WOMAC), ↓Stiffness*, and ↓Disability*	HILT and LLLT combined with exercise significantly reduced VAS and WOMAC scores after treatment, with HILT proving more effective than LLLT. Both resources outperformed placebo.	None	Institute of Scientific Research and Revival of Islamic Heritage at Umm Al-Qura University, Makkah, Saudi Arabia.
Alayat (2017) [37] Egypt	CNP	7/10	75 (M 75, F 0) 45.2 (6.1) years Dropout 0	EG (25): HILT + EX CG (25): LLLT + EX PG (25): HILT placebo + EX	2s/week for 6 weeks	(A) PI (VAS) (B) Disability (NDI)	T0: baseline T1: post-treatment (6 weeks)	EG: ↓PI* and ↓disability* CG: ↓PI* and ↓disability*	HILT is an effective treatment for CNP. When combined with exercise, HILT was more effective in reducing pain and improving function than LLLT or placebo.	None	No funding was received for this study
Taradaj (2018) [38] Poland	Lumbar disc degenerative changes	8/10	68 (M 36, F 32) 45.0 (7.1) years Dropout 0	EG (18): HILT PG1 (17): HILT placebo CG (16): LLLT PG2 (17): LLLT placebo	5s/week for 3 weeks	(A) PI (VAS) (B) PI (LQIP) (C) Disability (ODI) (D) Disability (RMQ) (E) ROM (Schober test) (D) ROM (LT)	T0: baseline T1: post-treatment (3 weeks) T2: follow-up (4 weeks) T3: follow-up (12 weeks)	EG: ↓PI*,↓disability*, and ↑ROM* PG1: ↓PI*, ↓disability*, and ↑ROM* CG: ↓PI*,↓disability*, and ↑ROM* PG 2: ↓PI*,↓disability*, and ↑ROM*	HILT and LLLT were ineffective for lumbar disc degeneration, showing no significant benefit over placebo. They did not reduce pain or improve mobility or function, and further studies are needed to clarify their efficacy	None	Ministry of Science and Higher Education as a part of statuary grant of the Wroclaw Medical University
Dekholsh (2018) [39] Iran	Knee OA	5/10	45 (M 45, F 0) 45.4 (7.0) years Dropout 0	EG (15): HILT + PT (TENS + US + EX + hot packs) CG (15): LLLT + PT (TENS + US + EX + hot packs) PG (15): HILT placebo + PT (TENS + US + EX + hot packs)	2s/week for 6 weeks	(A) PI (VAS) (B) Disability (WOMAC)	T0: baseline T1: post-treatment (6 weeks) T2: follow-up (6 weeks)	EG: ↓PI* and ↓disability* CG: ↓PI* and ↓disability* PG: w/o changes in PI and disability	HILT and LLLT showed similar effects. However, LLLT is considered more appropriate due to its greater cost-effectiveness for both therapists and patients	None	NS
Ordahan (2018) [40] Turkey	Plantar fasciitis	8/10	75 (M 15, F 60) 48.7 (11.1) years Dropout 5	EG (37): HILT + stretching + insole CG (38): LLLT + stretching + insole	3s/week for 3 weeks	(A) PI (VAS) (B) Heel sensivity (HTI) (C) PI (FAOS) (D) Symptoms (FAOS) (E) DLA (FAOS) (F) Sports (FAOS) (G) QoL (FAOS)	T0: baseline T1: post-treatment (3 weeks)	EG: ↓PI*, ↓Heel sensivity*, ↑ PI* (FAOS), ↑ Symptoms*, ↑DLA*, ↑Sports*, and ↑QoL* CG: ↓PI*, ↓Heel sensivity*, ↑ PI* (FAOS), ↑ Symptoms*, ↑DLA*, ↑Sports*, and ↑QoL*	HILT and LLLT reduced pain and disability in lumbar disc degeneration but were not superior to placebo. Further studies are needed to clarify their efficacy.	None	NS
Fekri (2019) [41] Iran	Lateral epicondylitis	5/10	30 (M NS, F NS) 49.5 (NS) years Dropout 0	EG (15): HILT + PT (TENS + US + ice + EX) CG (15): LLLT + PT (TENS + US + ice + EX)	5s/week for 2 weeks	(A) PI (VAS) (B) PPT (ALG) (C) Grip strength (DNM)	T0: baseline T1: post-treatment (2 weeks)	EG: ↓PI*, ↑ PPT*, and ↑Grip strength* CG: ↓PI*, ↑ PPT*, and ↑Grip strength*	HILT and LLLT, combined with physiotherapy, effectively reduced pain and tenderness and improved grip strength in tennis elbow, with no significant differences between the two.	None	NS
Abdelbasset (2020) [42] Saudi Arabia	Chronic nsLBP	7/10	60 (M 42, F 18) 32.9 (4.1) years Dropout 0	EG (20): HILT + EX (home) CG1 (20): LLLT + EX (home) CG2 (20): EX (home)	2s/week for 12 weeks	(A) PI (VAS) (B) ROM (Shober test) (C) Disability (ODI) (D) QoL (EQ-5D-3 L)	T0: baseline T1: post-treatment (12 weeks)	EG: ↓PI*, ↓disability*, ↑ROM*, and ↑QoL* CG: ↓PI*, ↓disability*, ↑ROM*, and ↑QoL* EG: ↓PI, ↓disability, ↑ROM (Shober test), and ↑QoL	LLLT and HILT show similar effects in treating chronic nsLBP, effectively reducing pain and disability while improving lumbar ROM and QoL.	None	Deanship of Scientific Research at Prince Sattam Bin Abdulaziz University
Sudiyono (2020) [43] Indonesia	CTS	5/10	16 (M 1, F 15) 48.0 (2.0) years Dropout 0	EG (8): HILT CG (8): LLLT	5s/week for 2 weeks	(A) EFGP: SNCV, DML, and CSI (EMG)	T0: baseline T1: post-treatment (2 weeks)	EG: ↑SNCV*, ↑DML, and ↓CSI* CG: ↑SNCV*, w/o changes in DML and CSI	HILT is more effective than LLLT for improving EFGP in moderate CTS. Future research should use larger, controlled samples and long-term evaluation.	None	Atma Jaya Catholic University of Indonesia
Hojjati (2020) [44] Iran	CTS	7/10	45 (M 0, F 45) 47.8 (1.9) years Dropout 0	EG1 (15): HILT + splint CG1 (15): LLLT + splint CG2 (15): splint	1s/week	(A) PI (VAS) (B) Pinch strength (DNM) (C) EFGP: SNAP latency, SNAP amplitude, CMAP OL, and CMAP amplitude (EMG) (D) Symptom severity (BCTQ-SS) (E) Disability (BCTQ-FS)	T0: baseline T1: post-treatment T2: follow-up (12 weeks)	EG: ↓PI*, ↓pinch strength*, ↑EFGPs*, ↓symptoms*, and ↓disability* CG1: ↓PI*, ↓pinch strength*, ↑EFGPs*, ↓symptoms*, and ↓disability* CG2: ↓PI, ↓pinch strength, ↑EFGPs, ↓symptoms, and ↓disability	Both wrist splints and laser therapy can improve CTS symptoms and function, with HILT showing slightly better results than LLLT therapy, though not significantly different.	None	NS
Ezzati (2020) [45] Iran	CTS	9/10	98 (M 16, F 82) 48.0 (8.9) years Dropout 0	EG1 (20): HILT (mild dose) + EX EG2 (19): HILT (high dose) + EX CG1 (20): LLLT (mild dose) + EX CG2 (19): LLLT (high dose) + EX CG3 (17): EX	2s/week for 3 weeks	(A) PI(VAS) (B) EFGP: CMAP amplitude, SNAP latency, and SNAP amplitude* (EMG)	T0: baseline T1: post-treatment (3 weeks)	EG1: ↓PI*, ↑CMAP amplitude*, ↓SNAP latency*, and SNAP amplitude* EG2: ↓PI*, ↑CMAP amplitude*, ↓SNAP latency*, and SNAP amplitude* CG1: ↓PI*, ↑CMAP amplitude*, ↓SNAP latency*, and SNAP amplitude* CG2: ↓PI*, ↑CMAP amplitude*, ↓SNAP latency*, and SNAP amplitude* EG1: ↓PI, ↑CMAP amplitude, ↓SNAP latency, and SNAP amplitude	Laser therapy is effective in treating CTS; both low-level and high-intensity laser therapies reduce pain and improve electrophysiologic indices. HILT at a mild dose showed the best results.	None	NS
Naruseviciute (2020) [46] Lithuania	Plantar fasciitis	7/10	102 (M 46, F 66) 56.2 (10.6) years Dropout 0	EG (51): HILT + PT (stretching + ice) + insole CG (51): LLLT + PT (stretching + ice) + insole	3s/week for 3 weeks	(A) PI (VAS) (B) PI (first steps and after walking) (VAS) (C) PPT (ALG) (D) Fascia thickness (USG)	T0: baseline T1: post-treatment (3 weeks) T2: follow-up (4 weeks)	EG: ↓PI*, ↓PI* (first steps and after walking), ↑PPT*, and ↓Fascia thickness* CG: ↓PI*, ↓PI* (first steps and after walking), ↑PPT*, and ↓Fascia thickness*	Both groups improved without significant differences between HILT and LLLT. Most participants found the treatments effective.	None	Lithuanian University of Health Sciences and Research Council of Lithuania.
Kaydok (2020) [47] Turkey	Lateral epicondylitis	9/10	60 (M 16, F 44) 44.2 (9.3) years Dropout 0	EG (30=: HILT + elbow bandage CG (30): LLLT + elbow bandage	3s/week for 3 weeks	(A) PI (VAS) (B) Disability (Q-DASH) (C) Grip strength (DNM) (D) QoL (SF-36)	T0: baseline T1: post-treatment (3 weeks)	EG: ↓PI*, ↓disability*, ↑grip strength, and ↑QoL* CG: ↓PI*, ↓disability*, ↑grip strength, and ↑QoL*	HILT and LILT effectively treated Lateral epicondylitis, with HILT showing a greater improvement in grip strength, QDASH, and SF-36.	None	No funding was received for this study
Zaki (2021) [48] Iran	SAIS	8/10	30 (M 13, F 17) 48.6 (12.8) years Dropout 0	EG (10): HILT + KT CG (10): LLLT + KT PG (10): HILT placebo + KT	3s/week for 2 weeks	(A) Changes in PI (VAS) (B) Changes in PI (SPADI) (C) Disability (SPADI) (D) Subacromial space (USG)	T0: baseline T1: post-treatment (2 weeks)	EG: ↑Changes in PI*, ↑Changes in disability*, and ↑Subacromial space* CG: ↑Changes in PI*, ↑Changes in disability*, and ↑Subacromial space* PG: w/o changes in PI*, disability*, and ↑Subacromial space*	HILT combined with KT showed greater improvements in pain, disability, and tendon echogenicity compared to LLLT with KT. Routine KT application also increased subacromial space, emphasizing its biomechanical benefits.	None	Tarbiat Modares University
Al-Kurdı (2022) [49] Turkey	Lumbar disc herniation	5/10	60 (M 36, F 24) 48.6 (NS) years Dropout 0	EG (20): HILT + EX CG (20): LLLT + EX PG (20): HILT placebo + EX	3s/week for 3 weeks	(A) PI (VAS) (B) ROM (Schober test) (C) Disability (ODI) (D) Disability (RMQ)	T0: baseline T1: post-treatment (3 weeks)	EG: ↓PI*, ↑ROM*, and ↓disability* CG: ↓PI*, ↑CROM*, and ↓disability* PG: ↓PI, ↑CROM, and ↓disability	Both HILT and LLLT were effective for lumbar disc herniation, with HILT demonstrating superior pain reduction, improved lumbar ROM, and faster recovery. Combining laser therapy with physical therapy prolonged the benefits.	None	NS
Abdelsalam (2022) [50] Egypt	Lumbar disc herniation	7/10	60 (M 25, F 35) 34.0 (5.6) years Dropout 0	EG (20): HILT + EX CG (20): LLLT + EX PG (20): HILT placebo + EX	3s/week for 4 weeks	(A) PI (VAS) (B) Disability (ODI) (C) SLR angle (degrees) (D) ROM (GNM)	T0: baseline T1: Intermediate evaluation (2 weeks) T2: post-treatment (4 weeks)	EG: ↓PI*, ↓disability*, ↑SLR*, and ↑ROM* CG: ↓PI*, ↓disability*, ↑SLR*, and ↑ROM* PG: ↓PI*, ↓disability*, ↑SLR*, and ↑ROM*	HILT is more effective than LLLT and placebo for treating acute and subacute lumbar disc herniation.	None	NS
Ahmad (2023) [51] Malaysia	Knee OA	9/10	34 (M 8; F 26) 54.6 (10.2) Dropout (0)	EG (17): HILT + PT (EX + MT) CG (17): LLLT + PT (EX + MT)	1s/week for 12 weeks	(A) PI (NPRS) (B) Pain (KOOS) (C) Symptoms (KOOS) (D) DLA (KOOS) (E) Sports (KOOS) (F) QoL (KOOS) (G) Function (KOOS-total) (H) ROM (GNM) (I) Disability (TUG)	T0: baseline T1: post-treatment (12 weeks)	EG: ↓PI*, ↓Symptoms*, ↑DAL*, ↑Sports*, ↑QoL*, ↑Function*, ↑ROM*, and ↓disability* CG: ↓PI*, ↓Symptoms*, ↑DAL*, ↑Sports*, ↑QoL*,↑Function*, ↑ROM*, and ↓disability*	Combining HILT or LLLT with PT significantly improved knee pain, disability, physical function, and mobility, with HILT showing greater benefits than LLLT.	None	Physiotherapy Program, Centre for Rehabilitation and Special Needs Studies, Faculty of Health Sciences, Universiti Kebangsaan Malaysia
Ordahan (2023) [52] Turkey	Frozen Shoulder	9/10	40 (M 9, F 31) 55.1 (8.4) years Dropout 0	EG (20): HILT + EX CG (20): LLLT + EX	3s/week for 3 weeks	(A) PI (VAS) (B) PI (SPADI) (C) ROM (GNM) (D) Disability (SPADI)	T0: baseline T1: post-treatment (3 weeks)	EG: ↓PI*, ↑ROM*, and ↓disability* CG: ↓PI*, ↑ROM*, and ↓disability*	A 3-week application of HILT is more effective than LLLT in reducing pain and improving function in patients with frozen shoulder.	None	No funding was received for this study
Astri (2023) [53] Indonesia	Knee OA	5/10	61 (M 4, F 57) 60.2 (7.2) years Dropout 0	EG (30): HILT + EX CG (31): LLLT + EX	3s/week for 2 weeks	(A) PI (VAS) (B) Function (50-feet walt test)	T0: baseline T1: during treatment (after each session) T2: post-treatment (3 weeks)	EG: ↓PI* (VAS) and ↑function* CG: ↓PI* (VAS) and ↑function*	HILT and LLLT significantly improved pain and function in knee OA, with HILT showing greater but not statistically significant benefits in VAS and walking speed.	None	NS
Rizky (2024) [54] Indonesia	Knee OA	5/10	27 (M 6, F 21) 60.1 (3.6) years Dropout 0	EG (14): HILT CG (13): LLLT	8 s (2s) 2s/week for 4 weeks	(A) PI (KOOS) (B) Symptoms (KOOS) (C) DLA (KOOS) (D) Sports (KOOS) (E) QoL (KOOS) (F) Function (KOOS-total)	T0: baseline T1: post-treatment (4 weeks)	EG: ↓PI*, ↓Symptoms*, ↑DAL*, ↑Sports*, ↑QoL*, and ↑Function* CG: ↓PI*, ↓Symptoms*, ↑DAL*, ↑Sports*, ↑QoL*, and ↑Function*	HILT could offer greater functional improvement than LLLT, especially for active patients with shorter treatment periods.	None	NS
Ezzati (2024) [55] Iran	Knee OA	7/10	98 (M NS, F NS) 50.4 (7.9) years Dropout 8	EG (30): HILT + PT (IR + TENS + stretching + EX) CG1 (30): LLLT + PT (IR + TENS + stretching + EX) CG2 (30): PT (IR + TENS + stretching + EX)	5s/week for 2 weeks	(A) Disability (WOMAC) (B) VMO and VL thickness (USG)	T0: baseline T1: post-treatment (2 weeks) T2: follow-up (4 weeks)	EG: ↓disability*, ↑VMO thickness*, and ↑VL thickness CG1: ↓disability*, ↑VMO thickness*, and ↑VL thickness CG2: ↓disability*, ↑VMO thickness*, and ↑VL thickness*	HILT and LLLT improve knee OA outcomes; HILT shows superior short-term efficacy. Further studies with objective measures are needed.	None	Deputy of Research and Technology of Guilan University of Medical Sciences
Şen (2025) [56]	Knee OA	8/10	90 (M 11, F 79) 52.4 (6.8) years Dropout 0	EG (45): HILT + EX CG (45): LLLT + EX	5s/week for 2 weeks	(A) PI (VAS) (B) Disability (WOMAC) (C) ROM (GNM) (D) FCT (USG)	T0: baseline T1: post-treatment (2 weeks) T2: follow-up (6 weeks)	EG: ↓PI*, ↓disability*, ↑ROM* CG: ↓PI*, ↓disability*, ↑ROM*	HILT and LLLT combined with exercise therapy improved pain, function, and cartilage thickness in patients with knee osteoarthritis, with HILT demonstrating superior therapeutic efficacy.	None	NS

Abbreviations: ALG- algometry; BBS- Berg Balance Scale; CG- control group; CMAP- compound muscle action potential; CNP- Chronic Neck Pain; CSI- combined sensory index; CTS- Carpal tunnel syndrome; DLA- daily life activities; DML- distal motor latency; DNM- dynamometry; EQ-5D-3L- EuroQoL 5-Dimension 3-Level; EFGP- electrophysiological parameters; EG- experimental group; EMG- electromyography; EX- exercise; FAOS- Foot and Ankle Outcome Score; FCT- femoral cartilage thickness; HILT- high-intensity laser therapy; HTI- Heel tenderness index; IR- infrared lamps; KT- kinesiotape; LBP- Low back pain; LLLT- low-level laser therapy; LQIP- Laitinen Questionnaire Indicators of Pain; LT- Lasegue test; MT- Manual Therapy; MSDs- musculoskeletal disorders; NDI- the Neck Disability Index; nsLBP- nsLBP; OA- Osteoarthritis; ODI- Oswestry disability index; OL- motor onset latency; PI- pain intensity; PG- placebo group; PPT- pain pressure threshold; QoL- Quality of life; NS- not specified; RMQ- Roland Morris Questionnaire; ROM- range of motion; SAIS- Subacromial impingement syndrome; SNAP- sensory nerve action potential; SNCV- sensory nerve conduction velocity; SPADI- Shoulder Pain and Disability Index; SLR- The straight leg raise test; TENS- transcutaneous electrical nerve stimulation; US- therapeutic ultrasound; USG- ultrasonography; VAS- Visual anolog scale; VL- Vastus lateralis muscle; VMO- vastus medialis obliquus muscle; WOMAC- Western Ontario and McMaster Universities Osteoarthritis Index; w/o- without. p < 0.05*

### Outcomes

 The studies primarily assessed pain intensity, disability, and range of motion (ROM). Pain intensity was most commonly measured using the Visual Analog Scale (VAS) [35–42, 44–53, 56] with additional measures including the Lattinen Index [35], the Numeric Pain Rating Scale (NPRS) [51], the WOMAC [36, 39, 55, 56], the Knee Injury and KOOS pain subscale [51] and the Shoulder Pain and Disability Index (SPADI) [47, 51]. Disability was evaluated using the ODI [38, 49, 50], the Neck Disability Index (NDI) [37], the Foot and Ankle Outcome Score (FAOS) [40],the Roland-Morris Questionnaire (RMQ) [38, 49], the Lequesne Index [35], the quick- DASH [47], and functional tests such as the Timed Up and Go (TUG) [51]. ROM was assessed using the Schober Test [38, 42] and goniometry [50–52, 56]. Less frequent outcomes included muscle strength [41, 44, 47], pressure pain threshold [41, 46], fascia thickness [46], subacromial space [48], electrophysiological parameters [43–45], vastus lateralis and medialis obliquus muscle thickness [55], and quality of life [40, 42].

 All trials included baseline and post-treatment assessments, with intervention periods ranging from 2 weeks [35, 41, 43, 48, 55, 56] to 12 weeks [42, 51]. Follow-up assessments were conducted in seven RCTs, encompassing periods of 3–4 weeks [46], 4 weeks [38, 55], 6 weeks [39, 56], and 12 weeks [38, 44]. No adverse events related to HILT or LLLT were reported in any of the included trials [35–56] (Table 1).

### Laser parameters

 Most HILT devices employed a wavelength of 1,064 nm [36, 38–40, 42–44, 46, 47, 49, 51–54, 56], while some studies used 808 nm [41, 45, 55], or devices combining wavelengths such as 808 and 905 nm [35, 37], or 810 and 980 nm [48]. Five studies used continuous emission [38, 39, 44, 46, 49, 55], five employed pulsed emission [36, 41, 45, 50], and eight incorporated both emission modes in two or more phases [35, 40, 43, 47, 48, 51, 53, 54, 56]. The peak power of HILT devices ranged from 1 to 3,000 W, with 12 W being the most common [38–44, 46, 47, 51, 53, 54, 56]. The mean power output across studies was 5.8 W (SD = 3.0). Thirteen RCTs employed a single-phase approach, of which twelve applied a scanning technique [38, 41, 42, 44, 45, 47–52] and four used a punctual (spot) technique [35, 39, 45, 50]. Additionally, five RCTs combined both scanning and punctual techniques [36, 37, 43, 46, 48, 56]. The total energy delivered per treatment ranged from 100 J in frozen shoulder [52] to 3,000–3,750 J in cases of frozen shoulder and plantar fasciitis [40, 52], with an average total dose between 1,000 and 1,200 J. For point-specific applications, the energy per point varied from 6.6 J in knee osteoarthritis [35] to 25 J in chronic neck pain [37], with an average of 19 J per point. In scanning applications, the energy delivered ranged from 150 J in lateral epicondylitis and plantar fasciitis to 3,750 J in plantar fasciitis [40], with a mean dose of 1,776 J. Treatment duration ranged from 36 seconds [44] to 900 seconds [42], with an average duration of 618 seconds. Other HILT parameters and specifications are summarized in Table 2.

**Table 2:** Characteristics and parameters of the lasers used in the studies

Laser type	Characteristics/Parameters	Gworys (2012) [35]	Kheshie (2014) [36]	Alayat (2017) [37]	Taradaj (2018) [38]	Dekholsh (2018) [39]	Ordahan (2018) [40]	Fekri (2019) [41]	Abdelbasset (2020) [42]	Sudiyono (2020) [43]	Hojjati (2020) [44]	Ezzati (2020) [45]	Naruseviciute (2020) [46]	Kaydok (2020) [47]	Zaki (2021) [48]	Al-Kurdı (2022) [49]	Abdelsalam (2022) [50]	Ahmad (2023) [51]	Ordahan (2023) [52]	Astri (2023) [53]	Rizky (2024) [54]	Ezzati (2024) [55]	Şen (2025) [56]
HILT	Laser model	Multiwave locked system (MLS) (ASA, Arcugnano, Italy)	HIRO 3.0 (ASA laser, Arcugnano, Italy)	Multiwave locked system (MLS) (ASA, Arcugnano, Italy)	Cyborg Laser (Cosmogamma, Jakarta, Indonesia)	BTL-6000 (BTL Industries, Prague, Czech Republic)	BTL-6000 (BTL Industries, Prague, Czech Republic)	MLS Laser M6 (ASA, Arcugnano, Italy)	BTL-6000 (BTL Industries, Prague, Czech Republic)	BTL-6000 (BTL Industries, Prague, Czech Republic)	BTL-6000 (BTL Industries, Prague, Czech Republic)	HC YAG Pagani Laser (Italy)	BTL-6000 (BTL Industries, Prague, Czech Republic)	BTL-6000 (BTL Industries, Prague, Czech Republic)	OptonPro Diode Laser (Zimmer, Germany)	OptonPro Diode Laser (Zimmer, Germany)	OptonPro Diode Laser (Zimmer, Germany)	BTL-6000 (BTL Industries, Prague, Czech Republic)	BTL-6000 (BTL Industries, Prague, Czech Republic)	BTL-6000 (BTL Industries, Prague, Czech Republic)	BTL-6000 (BTL Industries, Prague, Czech Republic)	HC YAG Pagani Laser (Italy)	BTL-6000 (BTL Industries, Prague, Czech Republic)
	Wavelength	808 nm and 905 nm	1,064 nm	808 nm and 905 nm	1,064 nm	1,064 nm	1,064 nm	808 nm	1,064 nm	1,064 nm	1,064 nm	808 nm	1,064 nm	1,064 nm	810 and 980 nm	1,064 nm	810 and 980 nm	1,064 nm	1,064 nm	1,064 nm	1,064 nm	808 nm	1,064 nm
	Mode (continuous/pulsed)	808 nm (continuous) 905 nm (pulsed)	Pulsed	808 nm (continuous) 905 nm (pulsed)	Continuous	Continuous	Pulsed (sessions 1–4) Continuous (sessions 5–10)	Pulsed	Continuous	Pulsed Continuous	Continuous	Pulsed	Continuous	Pulsed Continuous	Pulsed Continuous	Continuous	Pulse	Pulsed Continuous	Pulsed (Phase 1) Continuous (Phase 2)	Pulsed (Phase 1) Continuous (Phase 2)	Pulsed (Phase 1) Continuous (Phase 2)	Continuous	Pulsed (Phase 1) Continuous (Phase 2)
	Output power (W)	1 W (continuous mode) 25 W (pulsed mode)	3,000 W (3 kW)	1 W (continuous mode) 25 W (pulsed mode)	13 W	12 W	12 W	75 W 3.3 W (3 sources of 25 W)	12 W	12 W	12 W	15 W	12 W	12 W	7 W	7 W	8 W	12 W	12 W	12 W	12 W	15 W	12 W
	Mean power (W)	1.1 W	3 W	0.5 W (continuous mode) 0.054 W (pulsed mode)	10 W	5 W	8 W (sessions 1–4) 6 W (sessions 5–10)	3.3 W (3 sources of 1.1 W)	12 W	Phase 1: 1 W Phase 2: 5 W	5 W	1.6 W	7 W	Phase 1: 8 W (session 1–3) Phase 2: 6 W (Session 4–9)	4 W	7 W	3 W	5 W	Phase 1: 8 W (session 1–3) Phase 2: 12 W (session 4–9)	Phase 1: 10 W Phase 2: 5 W	Phase 1: 10 W Phase 2: 5 W	1.6 W	Phase 1: 4 W (sessions 1–5) Phase 2: 12 W (Sessions 6–10)
	Frequency (Hz) or duty cycle (%)	2,000 Hz	10–40 Hz (0.1%)	1,500 Hz	0 Hz (100%)	0 Hz (100%)	25 Hz (25%) (sessions 1–4) 0 Hz (100%) (sessions 5–10)	700 Hz	0 Hz (100%)	10–40%	0 Hz (100%)	10 Hz	0 Hz (100%)	Phase 1: 25 Hz (25%) Phase 2: 0 Hz (100%	Phase 1 and 3: 100% Phase 2: 50%	25 Hz	5 Hz (50%)	Phase 1: 25 Hz (25%) Phase 2: 0 Hz (100%	Phase 1: 25 Hz (25%) Phase 2: 0 Hz (100%	Phase 1: 25 Hz (80%) Phase 2: 0 Hz (100%	Phase 1: 25 Hz (80%) Phase 2: 0 Hz (100%	10 Hz	Phase 1: 25 Hz (80%) Phase 2: 0 Hz (100%
	Spot size (cm2 or diameter)	3.14 cm2	0.2 cm2	3.14 cm2	NS	NS	3.14 cm2	6.25 cm2	3.14 cm2	3.14 cm2	3.14 cm3	NS	3.14 cm3	3.14 cm4	0.8 cm2	1 cm2	1 cm2	3.14 cm2	3.14 cm2	3.14 cm2	3.14 cm2	NS	3.14 cm2
	Energy density (J/cm2)	6.21 J/cm2 (high dose) 3.28 J/cm2 (mild dose)	0.61 to 0.81 J/cm3	4 J/cm^2^	60 J/cm2	NS	6 J/cm2 (sessions 1–4) 120–150 J/cm2 (sessions 5–10)	13.9 J/cm2	150 J/cm2	Phase 1: 10 J/cm2 Phase 2: 120 J/cm2	20 J/cm2	8 and 20 J/cm2	120 J/cm2	Phase 1: 6 J/cm2 Phase 2: 120–150 J/cm2	20 J/cm2	50 J/cm2	6.4 J/cm2	19–150 J/cm2	Phase 1: 10 J/cm2 (session 1–3) Phase 2: 120 J/cm2 (session 4–9)	Phase 1: 12 J/cm2 Phase 2: 120 J/cm2	Phase 1: 12 J/cm2 Phase 2: 120 J/cm2	12 J/cm2	Phase 1: 12 J/cm2 (sessions 1–5) Phase 2: 120 J/cm2/sessions 6–10)
	Total energy (J)	12.4 J/point 6.6 J/point	Phase 1: 500 J Phase 2: 250 J (25 J/point) Phase 3: 500 J Total = 1,250 J	Phase 1: 300 J Phase 2: (25 J/point) Total = 2,250 J	1,200 J	1,400 J	150 J (sessions 1–4) 3,000–3,750 J (sessions 5–10)	272.4 J	1,200 J	NS	720 J	NS	3,000 J	Phase 1: 150 J (session 1–3) Phase 2: 900 J (session 4–9)	Phase 1: 1,000 J Phase 2: 50 J Phase 3: 1,000 J Total = 2,050 J	1,200 J	NS	3,190 J	100 J (session 1–3) 120 J (session 4–9)	300 J (Phase 1) 300 J (Phase 2)	300 J (Phase 1) 300 J (Phase 2)	480 J	300 J (Phase 1) 3,000 J (Phase 2)
	Treatment area (cm2) or number of points	11 points	Scanning area NS 5 points	75 cm2	30 cm2	4 points	25 cm2	NS	25 cm2	NS	NS	10 points	25 cm2	NS	Phase 1: 100 cm2 Phase 2: 0.8 cm2 Phase 3: 100 cm2	24 cm2	NS	20 cm2	8 points	25 cm2	25 cm2	20 cm2	25 cm2
	Treatment time (sec)	2 min 4 s (high dose) 1 min 6 s (mild dose)	15 min	Phase 1: 4 min and 16 s Phase 2: 240 s Total = 8 min and 16 s	10 min	4 min	1 min 45 s	3 min	15 min	NS	36 s	1 min 40 s (retinaculum) 4 min and 10 s (wrist)	7 min 8 s	Phase 1: 75 s (session 1–3) Phase 2: 30 s (session 4–9)	Phase 1: 30 s Phase 2: 12.5 s Phase 3: 60 s	2 min 48 s	13 s per point	10 min 38 s	1 min 15 s (session 1–3) 30 s (session − 9)	2 min (Phase 1) 4 min (Phase 2)	2 min (Phase 1) 4 min (Phase 2)	300 s	1 min 15 s (sessions 1–5) 4 min 10 s (sessions 6–10)
	Treatment protocol	3 points: medial/lateral aspect of the patellofemoral joint; 2 points: superior/inferior patellofemoral joint; 2 points in the popliteal fossa	Phase 1: knee scanning Phase 2: Application to 5 points (medial and lateral tibial condyles) Phase 3: knee scanning	Phase 1: scanning of neck extensors, sternocleidomastoid, and trapezius. Phase 2: application to eight neck trigger points.	Manual scan in the lumbar region	Punctual technique in anterior and inside of the knee joint	Manual scan in the plantar fascia	Scanning on the lateral epicondyle and the origin wrist extensor muscles	Scanning technique on the lumbar region	Phase 1: Punctual on the flexor retinaculum Phase 2: Scanning along median nerve course	Scanning on the flexor retinaculum	6 points on the retinaculum and 4 on the wrist	Phase 1: Slow scanning on plantar fascia Phase 2: Fast scanning enthesis zone and heel	Scanning technique on lateral surface of elbow for both phases	Phase 1: Scanning Phase 2: Punctual technique on subacromial space Phase 3: Scanning	Scanning technique	Punctual technique on the lumbar region	Scanning technique on anteromedial and anterolateral knee surfaces	Scanning technique parallels the joint line.	Scanning technique to medial and lateral knee joint space	Scanning technique to medial and lateral knee joint space	The scanning technique anterior, medial and lateral knee joint space	Applications were performed using continuous circular movements in the knee.
LLLT	Laser model	NS	BTL-5000 cluster laser (BTL Industries, Prague, Czech Republic)	BTL-5000 cluster laser (BTL Industries, Prague, Czech Republic)	LAS Expert (Physiomed Elektromedizin, Schnaittach, Germany)	NS	Diode Laser (Chatanooga Group, USA)	ALI70 Laser (Novin Company)	Diode Laser (Chatanooga Group, USA)	Endolaser 422 (Enraf Nonius, Netherlands)	Lumx 2 Laser (Fisioline, Italy)	Lasermed 4098 (CARCI, Brazil)	LAS-Expert (Physiomed Elektromedizin, Schnaittach, Germany)	Diode Laser (Chatanooga Group, USA)	OptonPro Diode Laser (Zimmer, Germany)	Diode Laser (Chatanooga Group, USA)	Diode Laser (Chatanooga Group, USA)	BTL-5825SL (BTL Industries, Prague, Czech Republic)	Diode Laser (Chatanooga Group, USA)	BTL-5110 (BTL Industries, Prague, Czech Republic)	EME Polyeter Evo Laser	Lasermed 4098 (CARCI, Brazil)	BTL-4000 smart laser (BTL Industries, Prague, Czech Republic)
	Wavelength	810 nm	830 nm	830 nm	785 nm	830 nm	904 nm	808 nm	850 nm	905 nm	775 nm	860 nm	785 nm	904 nm	810 and 980 nm	904 nm	850 nm	830 nm	904 nm	830 nm	905 nm	860 nm	830 nm
	Mode (continuous/pulsed)	Continuous	Pulsed	Pulsed	Continuous	Pulsed	Pulsed	Pulsed	Pulsed	Pulsed	Pulsed	Pulsed	Pulsed	Pulsed	Pulsed	Pulsed	Pulsed	Pulsed	Pulsed	Continuous	Continuous	Pulsed	Pulsed
	Output power (W)	0.4 W	0.8 W (0.1 W per probe)	0.8 W (0.1 W per probe)	0.065 W	0.5 W	0.5 W	0.1 W	0.8 W	0.1 W	45 W	NS	1.05 W (0.05 W per diode)	0.24 W	7 W	0.5 W	0.8 W	0.4 W	0.24 W	0.4 W	0.0 78 W	NS	0.8 W
	Mean power (W)	0.4 W	0.64 W	0.1 W	0.065 W	0.3 W	0.16 W	0.05 W	0.64 W	0.025 W	0.13 W	0.05 W	0.025 W per diode (21 diodes)	0.16 W	0.2 W	0.05 W	0.2 W	NS	0.16 W	0.4 W	0.0 78 W	0.05 W	0.64 W
	Frequency (Hz) or duty cycle (%)	0 Hz	1,000 Hz and 80%	100 Hz	0 Hz	NS	5,000 Hz	250 Hz	1,000 Hz (80%)	25%	6,500 Hz	NS	50–60 Hz (50%)	5,000 Hz	2%	5,000 Hz	1,000 Hz (80%)	NS	5,000 Hz	0 Hz (100%)	0 Hz (100%)	NS	1,000 Hz (80%)
	Spot size (cm2 or diameter)	NS	NS	0.25 cm2 (probe area)	0.1 cm2	0.028 cm2	1.5 cm2	NS	1 cm2	NS	NS	0.19 cm2	NS	0.5 cm2	0.8 cm2	1,5 cm2	1 cm2	1 cm2	0.5 cm2	1 cm2	1 cm2	0.19 cm2	NS (clúster probe)
	Energy density (J/cm2)	12.7 J/cm2	50 J/cm2	4 J/cm2	8 J/cm2	50 J/cm2	8.4 J/cm2	8 J/cm2	50 J/cm2	6 J/cm2	20 J/cm2	8 and 20 J/cm2	4 J/cm2	2.4 J/cm2	20 J/cm2	1.8 J/cm2	NS	10–12 J/cm2	3 J/cm2	10 J/cm2	1.5 J/cm2	12 J/cm2	50 J/cm2
	Total energy (J)	8 J/point	1,250 J	300 J	240 J	NS	640.4 J	8 J/point	1,200 J	NS	NS	NS	140 J	216 J	2,000 J	12 J (3 J/point)	NS	400 J	27 J	60 J	9 J	46 J	1,250 J
	Treatment area (cm2) or number of points	11 points	NS	8 points	NS	4 points	4.5 cm2	4 points	NS	NS	NS	10 points	70 cm2	6 points	10 points	4 points	13 points	20 cm2	9 points	NS	6 points	10 points	NS
	Treatment time	3 min 40 s	32 min 33 s	7 min 49 s	8 min	4 min	2 min 35 s	4 min	30 min	NS	36 s	32 s (retinaculum) 1 min 6 s (wrist)	6 min 40 s	3 min	16 min 40 s	4 min	NS	16 min 40 s	7 min 30 s	2 min 30 s	2 min	15 min 20 s	33 min 20 s
	Treatment protocol	3 points: medial/lateral aspect of the patellofemoral joint; 2 points: superior/inferior patellofemoral joint; 2 points in the popliteal fossa	Cluster laser in Direct contact and perpendicular to knee	Application to eight neck trigger points.	Manual scan in the lumbar region	Punctual technique in anterior and inside of the knee joint	Punctual tendon insertion (calcaneus) and 2 points on the medial border of the fascia.	Punctual technique on the lateral epicondyle and the origin wrist extensor muscles	Manual scan in the lumbar region	Phase 1: Punctual on the flexor retinaculum and along median nerve course	Punctual to Flexor retinaculum	6 points on the retinaculum and 4 on the wrist	Phase 1: Slow scanning on plantar fascia Phase 2: Fast scanning enthesis zone and heel	Punctual application on lateral side of elbow	Punctual technique on subacromial space	Punctual technique in the lumbar region	Punctual technique on the lumbar region	Scanning technique on anteromedial and anterolateral knee surfaces	Punctual technique on shoulder surface	Scanning technique to medial and lateral knee joint space	Punctual technique on medial and lateral knee joint space	Punctual technique on medial, lateral and anterior knee joint	

Hz, hertz; HILT- High-intensity laser therapy; J- Joules; LLLT- low-level laser therapy; min- minutes; NS- not specified; sec- seconds; W- Watt

 For LLLT, the wavelengths fell within the infrared spectrum, ranging from 775 nm [44] to 905 nm [42, 53], with 810 nm [35, 41, 48] and 830 nm being the most frequently applied [36, 37, 39, 51, 53, 56]. Additionally, one study stood out for using combined wavelengths of 810 and 980 nm [48]. Fifteen studies employed pulsed emission [36, 37, 39, 40, 44–52, 55, 56], while four studies used continuous emission [35, 38, 53, 54]. The peak power of LLLT devices for a single probe ranged from 0.065 W [38] to 45 W [44], with 0.4 to 0.8 W being the most reported peak powers [35, 39, 40, 42, 49–51, 53, 56]. Three studies employed cluster-type laser devices with maximum emission powers ranging from 0.8 W to 1.05 W [36, 37, 46, 56]. The average power output of the laser systems varied from 0.025 W in the treatment of carpal tunnel syndrome [43] to 0.64 W in studies addressing knee osteoarthritis and lumbar disc herniation [36, 42, 56], with 0.05 W being the most used setting [28, 41, 45, 49, 55].

 The punctual technique was the predominant method reported [35–37, 39–41, 45, 47–50, 52, 55, 56], whereas only seven studies applied scanning techniques exclusively [38, 42, 46, 51, 53]. The total energy delivered per treatment session varied between 9 J and 2,000 J, with 300 J being the most frequently administered dose. In point application protocols, the energy per point ranged from 1.5 J in knee OA [54] to 36 J in lateral epicondylitis and chronic neck pain [47], with a mean dose of approximately 8 J per point. For scanning applications, the total energy delivered ranged from 60 J [51] to 1,200 J [42]. A summary of additional LLLT parameters is presented in Table 2.

### Meta-analysis

**Fig. 3:**
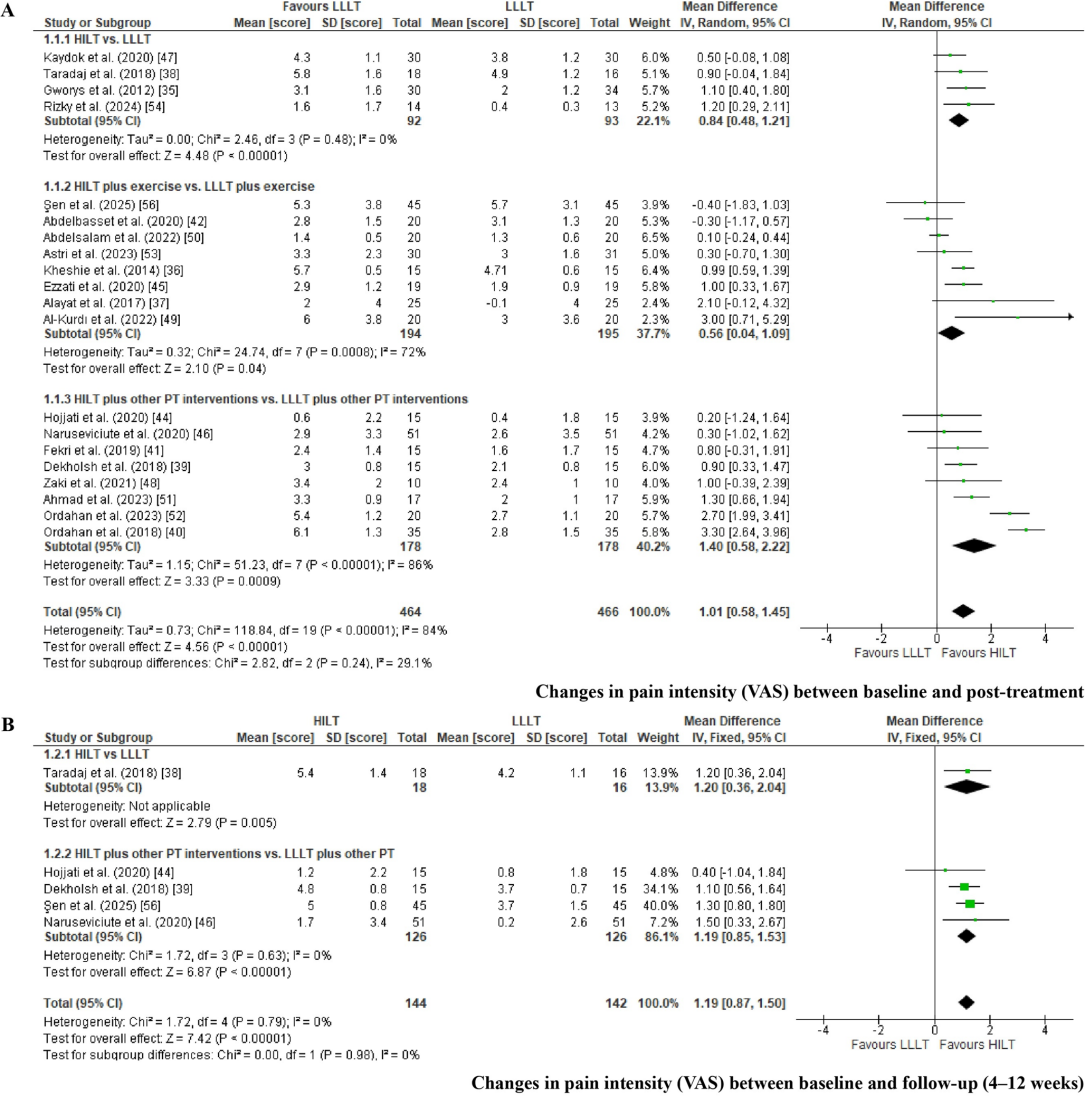
Forest plot for changes in pain intensity (VAS) between baseline and post-treatment (Fig. 3A): comparison of HILT versus LLLT applied alone (Fig. 1.1.1); HILT combined with therapeutic exercises versus LLLT with exercise (Fig. 1.1.2); and HILT versus LLLT, each combined with other physiotherapeutic interventions (TENS, ultrasound, cryotherapy, hot packs, manual therapy, kinesiology taping, and/or stretching) (Fig. 1.1.3). Forest plot for changes in pain intensity (VAS) between baseline and follow-up (4–12 weeks) (Fig. 3B): comparison of HILT versus LLLT applied alone (Fig. 1.2.1); and HILT versus LLLT, each combined with other physiotherapeutic interventions (TENS, ultrasound, cryotherapy, hot packs, manual therapy, kinesiology taping, and/or stretching) (Fig. 1.2.2).

*Change in pain intensity between baseline and post-treatment. *The meta-analysis for pain intensity included 20 RCTs categorized into three subgroups (Fig. 3A). The subgroups were as follows: HILT versus LLLT applied alone [35, 38, 47, 54]; HILT versus LLLT with both interventions combined with exercise therapy [36, 37, 42, 45, 49, 50, 53, 54, 56]; and HILT versus LLLT delivered in conjunction with additional physical therapy modalities, including TENS, ultrasound, cryotherapy, hot packs, stretching programs, kinesiology taping (K-tape), and insoles [39–41, 46, 48, 51]. Changes in pain intensity between baseline and post-treatment were analyzed using the VAS [35–42, 44–51, 53, 56], NPRS [51], and the KOOS pain subscale [54] as common outcome measures, and results were synthesized using MD. For three studies in which mean and standard deviation values were not reported [49, 53, 56], these parameters were estimated using the method proposed by Wang et al. [22]. Two studies were excluded from the analysis as they did not report pain intensity outcomes [43, 55]. A random-effects model was applied due to the observed heterogeneity (I² > 50%) [26].

 A statistically significant reduction in pain intensity favored HILT over LLLT when applied as a standalone intervention (MD = 0.84; 95% CI: 0.5 to 1.2; EG [n = 92], CG [n = 93]; p < 0.01), when both laser modalities were combined with exercise (MD = 0.56; 95% CI: 0.04 to 1.1; EG [n = 194], CG [n = 195]; p = 0.04), and when combined with other physical therapy interventions (MD = 1.4; 95% CI: 0.6 to 2.2; EG [n = 178], CG [n = 178]; p < 0.01). The overall pooled analysis also demonstrated a significant mean difference in favor of HILT (MD= 1.01; 95% CI: 0.6 to 1.5; EG [n = 464], CG [n = 466]; p < 0.01). When interpreting the magnitude of the observed effects, pain reduction was not considered clinically relevant for the comparison between HILT and LLLT applied alone, nor for the comparison in which both interventions were combined with exercise, as the MDs fell below the predefined threshold for clinical relevance (<10%) [28]. In contrast, the comparison between HILT and LLLT delivered alongside additional physical therapy modalities showed a clinically relevant effect, with MDs falling within the 10–20% range [28]. Across all comparisons, the certainty of the evidence was rated as very low according to the GRADE approach, primarily due to RoB, indirectness, heterogeneity, and imprecision (Table 3) [33, 34].

**Table 3:** Summary of findings and certainty of evidence (GRADE) for statistically significant outcomes

Certainty assessment							№ of patients			Certainty (Level of evidence)	Importance^f^
Studies	Study design	Risk of bias	Inconsistency	Indirectness	Imprecision	Publication bias	HILT	LLLT	Absolute (MD or SMD)		
									(95% CI)		
**Changes in pain intensity between baseline and post-treatment: HILT versus LLLT (VAS and KOOS pain section)**											
4 [35, 38, 47, 54]	RCT	serious^a^ (−1)	serious^b^ I^2^ = 0% (0)	serious^c^ (−1)	serious^d^ (−1)	no (0)	92	93	**MD = 0.8**	⨁◯◯◯ Very Low	Critical
									(0.5 to 1.2 greater)		
**Changes in pain intensity from baseline to post-treatment: HILT plus exercise versus LLLT plus exercise (VAS and KOOS pain section)**											
8 [36, 37, 42, 45, 49, 50, 53, 56]	RCT	not serious^a^ (−1)	serious^b^ I^2^ = 72% (−1)	serious^c^ (−1)	serious^d^ (−1)	no (0)	194	195	**MD = 0.6**	⨁◯◯◯ Very Low	Critical
									(0.04 to 1.1 greater)		
**Changes in pain intensity between baseline and post-treatment: HILT plus other PT interventions vs. LLLT plus other PT interventions (VAS and KOOS pain section)**											
8 [39–41, 44, 46, 48, 51, 52]	RCT	serious^a^ (−1)	serious^b^ I^2^ = 86% (−1)	serious^c^ (−1)	serious^d^ (−1)	no (0)	178	178	**MD = 1.4**	⨁◯◯◯ Very Low	Critical
									(0.6 to 2.2 greater)		
**Changes in pain intensity between baseline and follow-up (4–12 weeks): HILT versus LLLT (VAS)**											
1 [38]	RCT	serious^a^ (−1)	serious^b^ I^2^ = NA (−1)	serious^c^ (−1)	serious^d^ (−1)	NA	18	16	**MD = 1.2**	⨁◯◯◯ Very Low	Critical
									(0.4 to 2.0 greater)		
**Changes in pain intensity between baseline and follow-up (4–12 weeks): HILT plus other PT interventions vs. LLLT plus other PT interventions (VAS)**											
4 [39, 44, 46, 56]	RCT	serious^a^ (−1)	serious^b^ I^2^ = 0% (0)	serious^c^ (−1)	serious^d^ (−1)	no (0)	126	126	**MD = 1.2**	⨁◯◯◯ Very Low	Critical
									(0.9 to 1.5 greater)		
**Changes in pain intensity from baseline to post-treatment: HILT versus LLLT adhering to the WALT protocol (VAS)**											
7 [37, 38, 41, 42, 47, 49, 50]	RCT	serious^a^ (−1)	serious^b^ I^2^ = 56% (−1)	serious^c^ (−1)	serious^d^ (−1)	no (0)	331	334	**MD = 0.5**	⨁◯◯◯ Very Low	Critical
									(0.03 to 1.1 greater)		
**Changes in pain intensity from baseline to post-treatment: HILT versus LLLT not adhering to the WALT protocol (VAS**,** NPRS**,** KOOS pain section)**											
12 [35, 36, 39, 46–48, 51, 53, 56]	RCT	serious^a^ (−1)	serious^b^ I^2^ = 76% (−1)	serious^c^ (−1)	not serious^d^ (0)	no (0)	286	289	**MD = 1.1**	⨁◯◯◯ Very Low	Critical
									(0.7 to 1.6 greater)		
**Changes in Pain Intensity from Baseline to Post-Treatment in Knee Osteoarthritis: Overall Comparison of HILT versus LLLT Protocols (VAS and KOOS Pain Subscale)**											
4 [35, 38, 47, 54]	RCT	serious^a^ (−1)	serious^b^ I^2^ = 76% (−1)	not serious^c^ (0)	serious^d^ (−1)	no (0)	152	157	**MD = 0.8**	⨁◯◯◯ Very Low	Critical
									(0.5 to 1.2 greater)		
**Changes in Pain Intensity from Baseline to Post-Treatment in Carpal Tunnel Syndrome: Overall Comparison of HILT versus LLLT Protocols (VAS)**											
2 [44, 45]	RCT	serious^a^ (−1)	serious^b^ I^2^ = 0% (0)	not serious^c^ (0)	serious^d^ (−1)	NA	34	34	**MD = 0.9**	⨁◯◯◯ Very Low	Critical
									(0.3 to 1.5 greater)		
**Changes in Pain Intensity from Baseline to Post-Treatment in Lateral Epicondylitis: Overall Comparison of HILT versus LLLT Protocols (VAS)**											
2 [41, 47]	RCT	serious^a^ (−1)	serious^b^ I^2^ = 0% (0)	not serious^c^ (0)	serious^d^ (−1)	NA	45	45	**MD = 0.6**	⨁⨁◯◯ Low	Critical
									(0.1 to 1.1 greater)		
**Changes in Pain Intensity from Baseline to Post-Treatment in Shoulder Disorders: Overall Comparison of HILT versus LLLT Protocols (VAS)**											
2 [48, 52]	RCT	serious^a^ (−1)	serious^b^ I^2^ = 78% (−1)	not serious^c^ (0)	serious^d^ (−1)	NA	30	30	**MD = 2.0**	⨁◯◯◯ Very Low	Critical
									(0.3 to 3.6 greater)		
**Changes in disability between baseline and post-treatment: HILT vs. LLLT (Lequesne Index**,** ODI**,** and Q-DASH)**											
3 [35, 38, 47]	RCT	serious^a^ (−1)	serious^b^ I^2^ = 57% (−1)	serious^c^ (−1)	serious^d^ (−1)	yes (−1)	78	80	**SMD = 0.6**	⨁◯◯◯ Very Low	Critical
									(0.1 to 1.1 greater)		
**Changes in disability between baseline and post-treatment: HILT plus exercise vs. LLLT plus exercise (ODI**,** BQ-FS**,** and total WOMAC)**											
8 [36, 37, 40, 42, 49, 50, 52, 56]	RCT	serious^a^ (−1)	serious^b^ I^2^ = 94% (−1)	serious^c^ (−1)	serious^d^ (−1)	yes (−1)	200	200	**SMD = 1.5**	⨁◯◯◯ Very Low	Critical
									(0.6 to 2.4 greater)		
**Changes in disability between baseline and follow-up (4–12 weeks): HILT vs. LLLT (ODI)**											
1 [38]	RCT	serious^a^ (−1)	serious^b^ I^2^ = NA (−1)	serious^c^ (−1)	serious^d^ (−1)	NA	18	16	**SMD = 1.4**	⨁◯◯◯ Very Low	Critical
									(0.6 to 2.1 greater)		
**Changes in disability between baseline and follow-up (4–12 weeks): HILT plus other PT interventions vs. LLLT plus other PT interventions (BQ-FS and total WOMAC)**											
3 [39, 44, 55]	RCT	serious^a^ (−1)	serious^b^ I^2^ = 93% (−1)	serious^c^ (−1)	serious^d^ (−1)	yes (−1)	60	60	**SMD = 1.9**	⨁◯◯◯ Very Low	Critical
									(0.1 to 3.7 greater)		
**Changes in ROM from Baseline to Post-Treatment: HILT Plus Exercise versus LLLT Plus Exercise (Goniometry)**											
4 [42, 50, 52, 56]	RCT	serious^a^ (−1)	serious^b^ I^2^ = 22% (0)	serious^c^ (−1)	serious^d^ (−1)	yes (−1)	105	105	**SMD = 0.4**	⨁◯◯◯ Very Low	Important
									(0.2 to 0.7 greater)		
**Changes in ROM between baseline and post-treatment: HILT plus other PT interventions vs. LLLT plus other PT interventions (Goniometry)**											
1 [47]	RCT	serious^a^ (−1)	serious^b^ I^2^ = NA (−1)	serious^c^ (−1)	serious^d^ (−1)	NA	17	17	**SMD = 0.8**	⨁◯◯◯ Very Low	Important
									(0.1 to 1.5 greater)		

Abbreviations: BQ-FS, Boston Carpal Tunnel Questionnaire – Functional Status Scale; CI, confidence interval; FAOS, Foot and Ankle Outcome Score; HILT, high-intensity laser therapy; LLLT, low-level laser therapy; MCID, minimal clinically important difference; MD, mean difference; NA, not assessable (limited study included); ODI, Oswestry Disability Index; PT, physical therapy; Q-DASH, Quick Disabilities of the Arm, Shoulder and Hand questionnaire; RCT, randomized controlled trial; SMD, standardized mean difference; WOMAC, Western Ontario and McMaster Universities Osteoarthritis Index

(a) High risk of bias was primarily related to outcome measurement, with an overall risk of bias of 54.5%, indicating a moderate level (>50%)

(b) Heterogeneity determined inconsistency, based on the I² statistic (≥50%)

(c) Indirectness was judged as serious because the evidence combined different clinical conditions, dosing parameters, and follow-up durations, reducing the direct applicability of the results to a specific musculoskeletal disorder

(d) Imprecision was assessed by examining the width of the 95% CI and the total sample size (n < 400). For outcomes analyzed using MDs, imprecision was judged based on whether the CI crossed the predefined MCID. For outcomes analyzed using SMDs, imprecision was evaluated based on whether the CI crossed the line of no effect (SMD = 0) or thresholds corresponding to small-to-moderate effect sizes [33, 34]

(e) Publication bias was assessed using funnel plots and Egger’s regression test for pain intensity and disability outcomes (>10 studies). No evidence of publication bias was observed for pain intensity (Egger’s intercept = 0.54, 95% CI −3.65 to 4.73; p = 0.802), whereas disability outcomes showed funnel plot asymmetry supported by Egger’s test (intercept = 8.68, 95% CI 5.09 to 12.28; p < 0.05). For outcomes reported in fewer than three studies, publication bias could not be formally assessed due to insufficient data [31]

(f) Outcome importance (not important, important, or critical) was defined a priori according to GRADE guidance, based on relevance for clinical decision-making [33, 34]

 The network meta-analysis (Fig. 7A) compared 15 treatment modalities across 20 RCTs (11 two-arm and 9 multi-arm designs), using changes in pain intensity measured by the VAS [35–42, 44–51, 53, 56], NPRS [51], and the KOOS pain subscale [54], with a mean pain change of 2.9. Two studies were excluded from the analysis because they did not report pain intensity outcomes [43, 55]. The model demonstrated an adequate fit (D̄ = 52.9; observed = 53), confirming network connectivity and showing no evidence of inconsistency (p > 0.05), despite substantial heterogeneity (τ ≥ 50%) [29, 30].

 The network comprised 1,189 participants, contributing to 30 direct pairwise comparisons and 105 potential indirect comparisons. The most influential contrasts—reflected by larger node sizes and thicker connecting edges—were observed between HILT and LLLT, HILT and LLLT combined with exercise, and HILT and LLLT combined with other physical therapy interventions (e.g., TENS, ultrasound, cryotherapy, heat therapy, manual therapy, kinesiology taping, insoles, and/or stretching). The Litmus-O-gram rank plot (Fig. 7B) indicated that mild-dose HILT (1–6 W; fluence 8 J/cm²) combined with exercise (SUCRA = 79.3%) and exercise alone (SUCRA = 78.7%) were the most effective interventions for pain reduction among laser-based treatments, whether applied alone or in combination with other therapies. In contrast, isolated placebo laser interventions (HILT or LLLT) showed the lowest probability of effectiveness (SUCRA = 13.7% for LLLT and 11.2% for HILT) [29, 30], although these conditions were evaluated in a limited number of studies [35, 38].

*Change in pain intensity between baseline and follow-up (4–12 weeks). *The meta-analysis included five RCTs that used VAS scores to quantify changes in pain intensity between baseline and follow-up assessments (4–12 weeks) [38, 39, 44, 46, 56] (Fig. 3B). The studies were subgrouped into two comparisons: HILT versus LLLT applied alone, and HILT versus LLLT combined with additional physical therapy interventions (e.g., TENS, ultrasound, exercise, hot packs, stretching with ice, splints, or insoles), all assessed using VAS. A fixed-effects model was applied due to the low heterogeneity observed (I² < 50%) [26]. Statistically significant differences favored HILT over LLLT both when applied alone (MD = 1.2; 95% CI 0.4 to 2.0; EG [n = 18], CG [n = 16]; p < 0.01) and when combined with other physical therapy interventions (MD = 1.2; 95% CI 0.9 to 1.5; EG [n = 126], CG [n = 126]; p < 0.01). At follow-up (4–12 weeks), the magnitude of the observed pain reduction was clinically relevant for the comparison between HILT and LLLT delivered alongside additional physical therapy interventions, as the mean difference fell within the predefined 10–20% range [28]. Across all follow-up comparisons, the certainty of the evidence was rated as very low according to the GRADE approach, primarily due to RoB, indirectness, heterogeneity, and imprecision (Table 3) [33, 34].

**Fig. 4:**
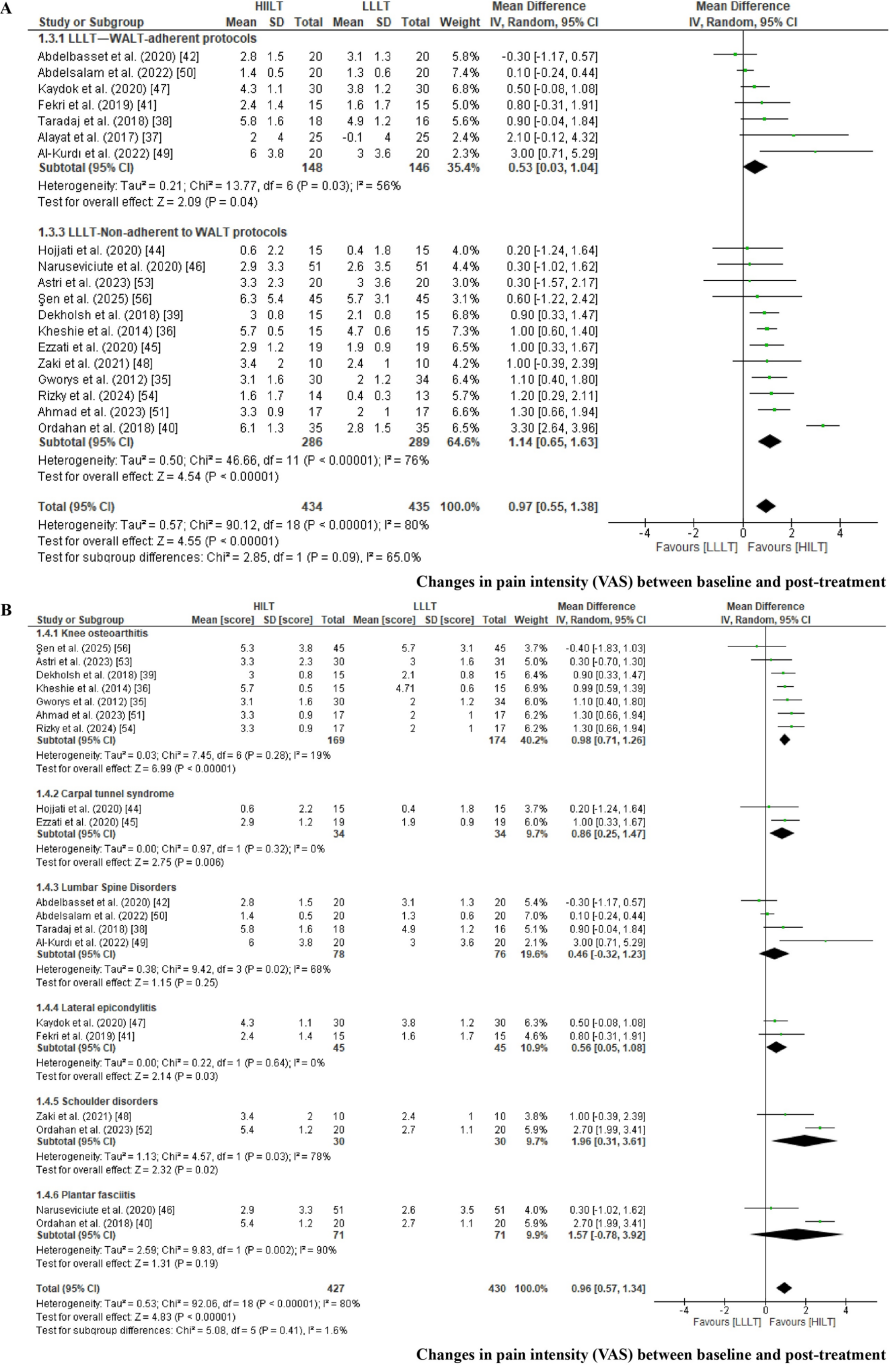
Forest plot for changes in pain intensity between baseline and post-treatment, grouped by adherence of LLLT protocols to the World Association for Laser Therapy (WALT) recommendations (Fig. 4A): LLLT—WALT-adherent protocols (1.3.1) and LLLT—not adherent to WALT (1.3.3). Forest plot for changes in pain intensity between baseline and post-treatment, grouped by musculoskeletal disorder (Fig. 4B): comparison of HILT versus LLLT in knee osteoarthritis (1.4.1), carpal tunnel syndrome (1.4.2), lumbar spine disorders (1.4.3), lateral epicondylitis (1.4.4), shoulder disorders (1.4.5), and plantar fasciitis (1.4.6)

*Change in pain intensity according to LLLT adherence to WALT recommendations*. Changes in pain intensity were analyzed according to whether LLLT protocols adhered to the dosage recommendations of the World Association for Laser Therapy (WALT). The meta-analysis included 19 RCTs categorized as WALT-adherent [37, 38, 41, 42, 47, 49, 50] or non-adherent [35, 36, 39, 41, 46, 48, 51, 53, 56] (Fig. 4A). No comparison was undertaken for frozen shoulder because WALT does not provide dosage guidance for this condition [52], and two trials were excluded from the analysis due to the absence of pain intensity outcomes [43, 55]. The pooled mean difference (MD) was calculated from changes in pain intensity between baseline and post-treatment using VAS [35–42, 44–51, 53, 56], NPRS [51], and the KOOS pain subscale [54]. A random-effects model was applied due to the substantial heterogeneity across comparisons (I² > 50%) [26]. Statistically significant differences favored HILT over LLLT for both subgroups. When HILT was compared with WALT-adherent LLLT protocols, a small effect was observed (MD = 0.53; 95% CI: 0.03 to 1.1; EG [n = 148], CG [n = 146]; p = 0.04). A larger effect was found when HILT was compared with non-adherent LLLT protocols (MD = 1.14; 95% CI: 0.7 to 1.6; EG [n = 286], CG [n = 289]; p < 0.05). When interpreting changes in pain intensity from baseline to post-treatment, the comparison between HILT and WALT-adherent LLLT did not demonstrate a clinically meaningful effect, as the mean difference did not reach the predefined 10% threshold for clinical relevance. In contrast, the comparison between HILT and non–WALT-adherent LLLT protocols showed a clinically relevant pain reduction, with mean differences falling within the 10–20% range []. For both comparisons, the certainty of the evidence was rated as very low according to the GRADE approach, primarily due to RoB, indirectness, heterogeneity, and imprecision (Table 3) [33, 34].

*Change in pain intensity between baseline and post-treatment across MSDs.* The meta-analysis included 19 RCTs grouped by pathology: knee osteoarthritis [35, 36, 39, 51, 53, 54, 56], carpal tunnel syndrome [44, 45], lumbar spine disorders [38, 42, 49, 50], lateral epicondylitis [41, 47], shoulder disorders [48, 52], and plantar fasciitis [40, 46] (Fig. 4B). Chronic neck pain was not included because it was reported in only one RCT [37], and two additional studies were excluded because they did not report pain intensity outcomes [43, 55]. The pooled MD was calculated based on the change in VAS scores [35–38, 42, 44–50, 52, 53, 56], NPRS scores [51], and the KOOS pain subscale [54] from baseline to post-treatment. A random-effects model was applied due to the heterogeneity observed across comparisons (I²> 50%) [26]. Statistically significant reductions in pain intensity favoring HILT were found for knee osteoarthritis (MD = 0.98; 95% CI 0.8 to 1.3; EG [n = 169], CG [n = 174]; p < 0.01), carpal tunnel syndrome (MD = 0.9; 95% CI 0.3 to 1.5; EG [n = 34], CG [n = 34]; p < 0.01), lateral epicondylitis (MD = 0.6; 95% CI 0.1 to 1.1; EG [n = 45], CG [n = 45]; p < 0.01), and shoulder disorders (MD = 2.0; 95% CI 0.3 to 3.6; EG [n = 30], CG [n = 30]; p < 0.01). No significant differences were observed for plantar fasciitis [40, 46] or lumbar spine disorders [38, 42, 49, 50]. When interpreting the magnitude of the observed effects, a clinically relevant pain reduction was identified only for shoulder disorders, as the mean differences fell within the predefined 10–20% range of clinical relevance. In contrast, for knee osteoarthritis, carpal tunnel syndrome, and lateral epicondylitis, the observed mean differences did not reach the 10% threshold and were therefore not considered clinically relevant [28]. According to the GRADE approach, the certainty of the evidence was rated as low for lateral epicondylitis and as very low for the remaining conditions—including knee osteoarthritis, carpal tunnel syndrome, shoulder disorders, and plantar fasciitis—primarily due to the limited number of available studies, imprecision, and concerns related to the overall RoB (Table3) [33, 34].

**Fig. 5:**
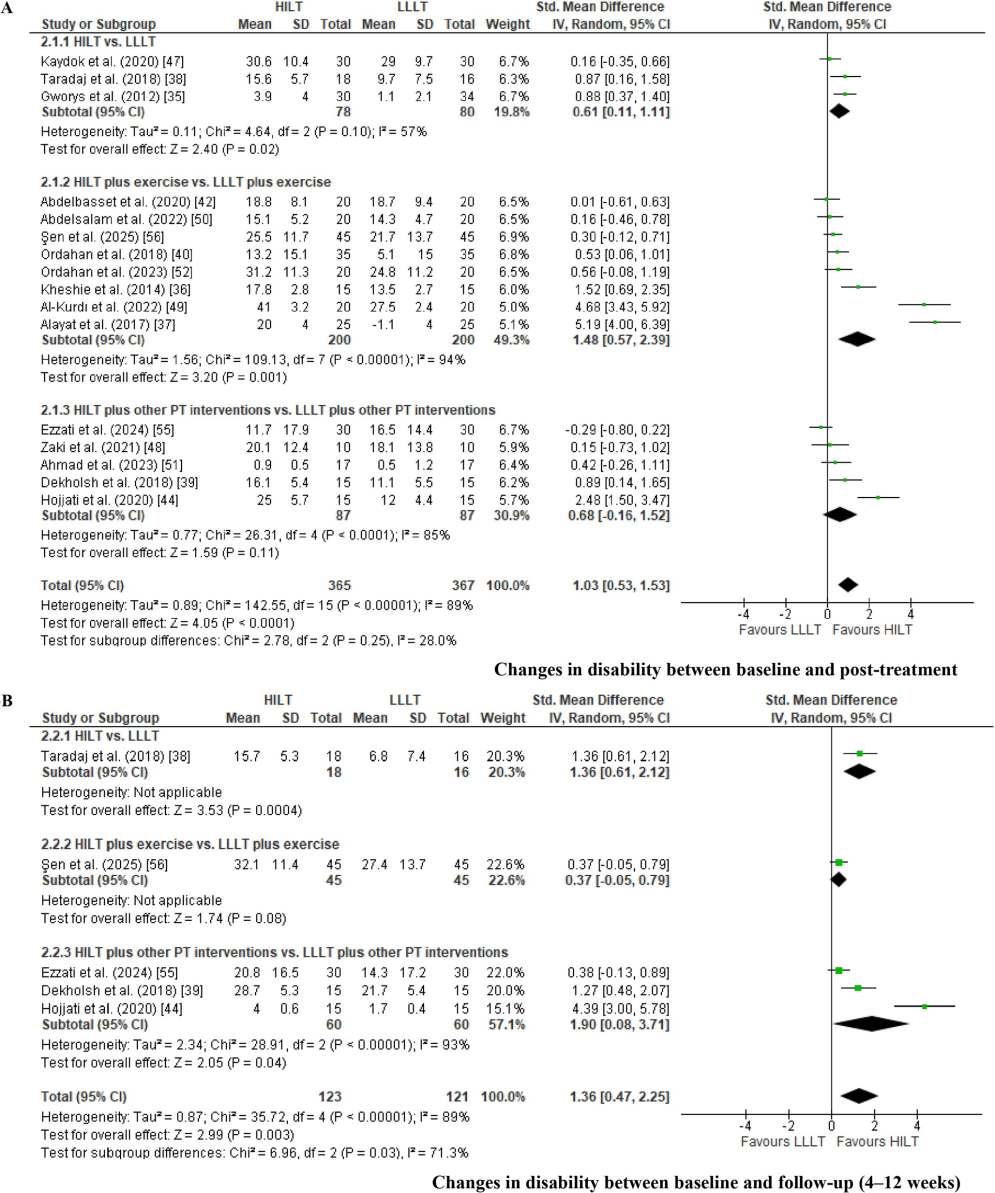
Forest plot for changes in disability between baseline and post-treatment (Fig. 5A): Comparison of HILT versus LLLT alone (Fig. 2.1.1); HILT combined with therapeutic exercises versus LLLT with exercise (Fig. 2.1.2); and HILT versus LLLT, both added to other physical therapy interventions (TENS, ultrasound, cryotherapy, heat therapy, manual therapy, kinesiology taping, and/or stretching) (Fig. 2.1.3). Forest plot for changes in disability between baseline and follow-up (4–12 weeks) (Fig. 5B): Comparison of HILT versus LLLT alone (Fig. 2.2.1); HILT combined with therapeutic exercises versus LLLT with exercise (Fig. 2.2.2); and HILT versus LLLT, both added to other physical therapy interventions (TENS, ultrasound, cryotherapy, heat therapy, manual therapy, kinesiology taping, and/or stretching) (Fig. 2.2.3).

*Change in disability between baseline and post-treatment*. The meta-analysis included 16 RCTs categorized into three comparison subgroups (Fig. 5A): both laser therapies applied alone [35, 38, 47]; both combined exclusively with exercise [36, 37, 40, 42, 50, 52, 56]; and both combined with other physical therapy interventions, such as TENS, ultrasound, exercise, hot packs, splinting, K-taping, manual therapy, infrared irradiation, or stretching [39, 44, 48, 51, 55]. Disability outcomes were assessed using several validated instruments, including the Lequesne Index [35], ODI [38, 42, 49], NDI [37, 50], BQ-FS [44], Q-DASH [47], WOMAC [36, 39, 55, 56], TUG [51], FAOS [40], and SPADI [48, 52], with changes from baseline to post-treatment expressed as SMDs. A random-effects model was applied due to significant heterogeneity (I² > 50%) [26]. A statistically significant and moderate effect size favored HILT over LLLT when applied alone (SMD = 0.6; 95% CI 0.1 to 1.1; EG [n = 78], CG [n = 80]; p = 0.02). A large effect size favored HILT combined with exercise compared with LLLT combined with exercise (SMD = 1.48; 95% CI 0.6 to 2.4; EG [n = 200], CG [n = 200]; p < 0.05). No significant differences were observed between HILT and LLLT when delivered as part of a multimodal physical therapy approach. When interpreting changes in disability from baseline to post-treatment, HILT compared with isolated LLLT demonstrated a clinically relevant improvement of moderate magnitude, corresponding to a moderate effect size (SMD = 0.5–0.8). In contrast, HILT compared with LLLT combined with exercise showed a clinically improvement of large magnitude (SMD ≥ 0.8) [27, 28]. According to the GRADE approach, the certainty of the evidence for both comparisons was rated as very low, primarily due to RoB, heterogeneity, indirectness, and imprecision (Table 3) [33, 34].

 Because disability outcomes were assessed using different measurement instruments across the included studies, all scores were normalized to a standardized 0–100 scale to ensure methodological consistency, improve comparability, and enable their inclusion in the network meta-analysis. The network meta-analysis (Fig. 7C) compared 11 treatment modalities across 15 randomized controlled trials (7 two-arm and 8 multi-arm designs), with a mean disability change of 16.8% across studies [35–40, 42, 44, 47, 48, 50, 51, 55, 56]. The model demonstrated an adequate fit (D̄ = 39.0; observed = 39), confirmed network connectivity, and showed no evidence of inconsistency (p > 0.05), despite the presence of substantial heterogeneity (τ ≥ 50%) [29, 30]. Overall, 831 participants were included, contributing to 18 direct pairwise comparisons and 55 potential indirect comparisons. The most clinically relevant contrasts were observed between HILT and LLLT when combined with therapeutic exercise, as well as between HILT and LLLT combined with other physical therapy interventions (e.g., TENS, ultrasound, cryotherapy, heat therapy, manual therapy, kinesiology taping, stretching, or insoles). The rank plot for disability outcomes (Fig. 7D) indicated that HILT combined with other physical therapy interventions (SUCRA = 76.7%) and HILT combined with exercise (SUCRA = 76.1%) had the highest probability of being the most effective strategies for improving disability when considering all laser-based interventions, whether applied alone or in combination with adjunctive therapies [29, 30]. In contrast, placebo laser (HILT or LLLT) combined with exercise showed the lowest probability of effectiveness (SUCRA = 19.8%), although the number of trials assessing this comparison for disability outcomes was limited.

Change in disability between baseline and follow-up (4–12 weeks). The meta-analysis included five RCTs grouped into three comparison subgroups [38, 39, 44, 47, 56] (Fig. 5B): HILT versus LLLT applied alone; HILT versus LLLT, each combined with exercise; and HILT versus LLLT combined with other physical therapy interventions, such as TENS, therapeutic ultrasound, exercise, hot packs, stretching with ice, splinting, or insoles. No statistically significant differences were observed between HILT plus exercise and LLLT plus exercise. Differences in disability between baseline and follow-up were expressed as SMDs derived from validated disability measures, including the ODI [37], BQ-FS [44], and total WOMAC [39, 55]. A random-effects model was applied due to the observed heterogeneity (I² > 50%) [26]. A statistically significant and moderate effect size (d = 0.5–0.8) favored HILT compared with LLLT when applied alone (SMD = 1.4; 95% CI: 0.6 to 2.1; EG [n = 18], CG [n = 16]; p < 0.01). A large effect size (> 0.8) also favored HILT compared with LLLT when both were combined with additional physiotherapeutic interventions (SMD = 1.9; 95% CI: 0.1 to 3.7; EG [n= 60], CG [n= 60]; p < 0.05) (Fig. 4B). When interpreting changes in disability from baseline to post-treatment at follow-up (4–12 weeks), both comparisons showed a clinically relevant improvement, corresponding to large effect sizes (SMD ≥ 0.8) [27, 28]. According to the GRADE approach, the certainty of the evidence for these comparisons was rated as very low due to heterogeneity, indirectness, risk of bias, publication bias, and imprecision (Table 3) [33, 34].

**Fig. 6:**
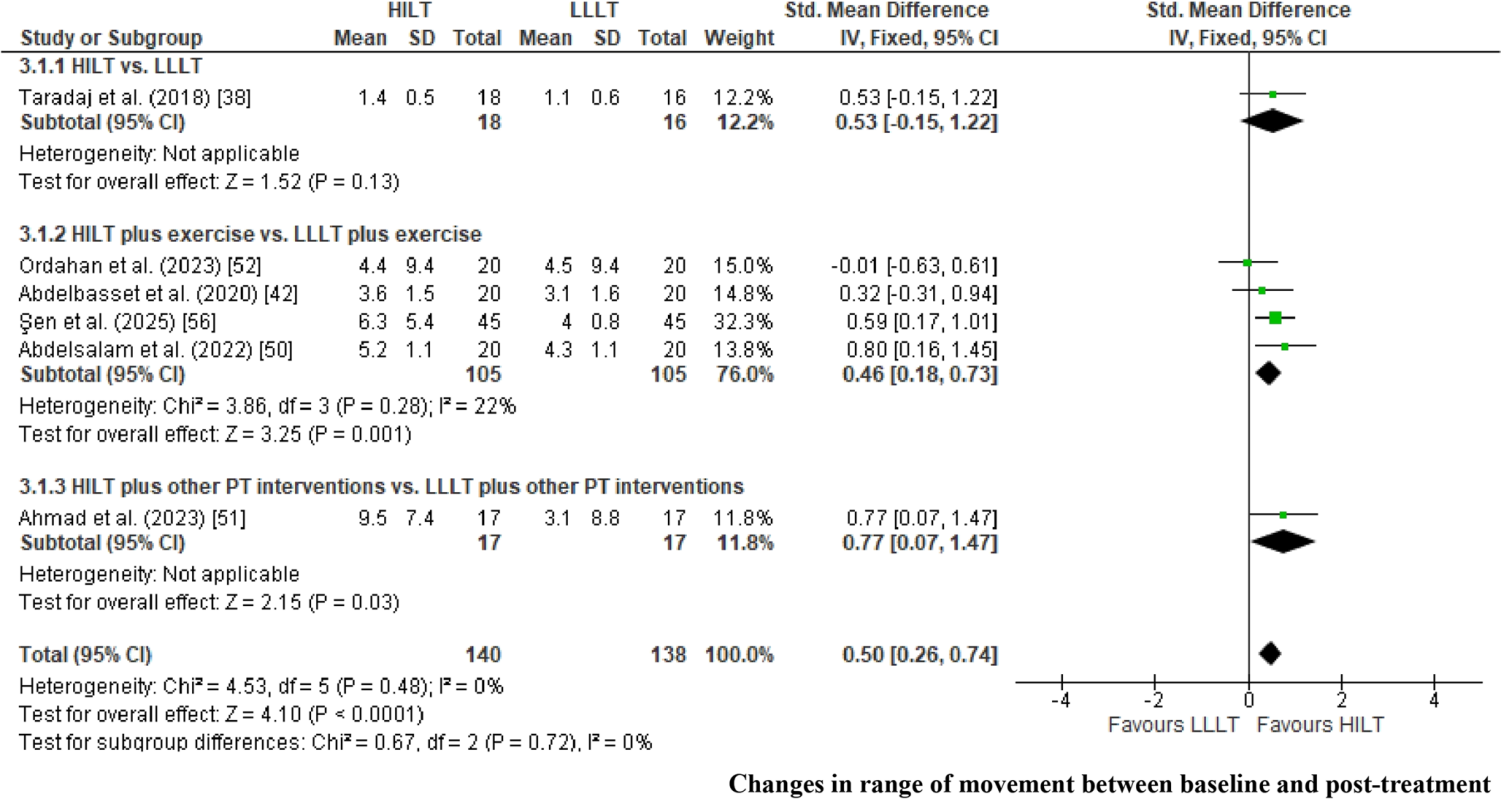
**Forest plot for changes in range of movement between baseline and post-treatment: **Comparison of HILT versus LLLT applied alone (Fig. 3.1.1); HILT combined with therapeutic exercises versus LLLT with exercise (Fig. 3.1.2); and HILT versus LLLT, each added to other physical therapy interventions (TENS, ultrasound, cryotherapy, hot packs, manual therapy, kinesiology tape, or stretching) (Fig. 3.1.3).

*Change in ROM between baseline and post-treatment. *The meta-analysis included six studies grouped according to the type of laser comparison (Fig. 6): HILT versus LLLT applied alone [39]; HILT versus LLLT [38], both combined with therapeutic exercises [42, 50, 52, 56]; and HILT versus LLLT, each integrated with other physical therapy interventions (manual therapy and therapeutic exercise) [51]. Outcomes related to joint mobility were analyzed based on changes from baseline to post-treatment using goniometric measures [53–56] and the Schober test [37, 41], expressed as SMDs. A fixed-effects model was applied due to the low observed heterogeneity (I² < 50%) [26]. A statistically significant moderate effect size was found for HILT compared with LLLT when both interventions were combined with additional physical therapy modalities (SMD = 0.77; 95% CI: 0.1 to 1.5; EG [n = 17], CG [n = 17]; p < 0.05). For HILT plus exercise versus LLLT plus exercise, statistically significant differences also favored HILT, with a moderate effect size (MD = 0.46; 95% CI: 0.2 to 0.7; EG [n = 105], CG [n = 105]; p < 0.01). When interpreting changes in ROM from baseline to post-treatment, the comparison between HILT and LLLT combined with other physical therapy interventions showed an improvement of small-to-moderate magnitude, with standardized mean differences ranging from 0.4 to 0.8, suggesting potential clinical relevance [27, 28]. According to the GRADE approach, the certainty of the evidence for this comparison was rated as very low due to RoB, heterogeneity, indirectness, imprecision, and the limited number of studies contributing data (Table 3) [33, 34].

**Fig. 7:**
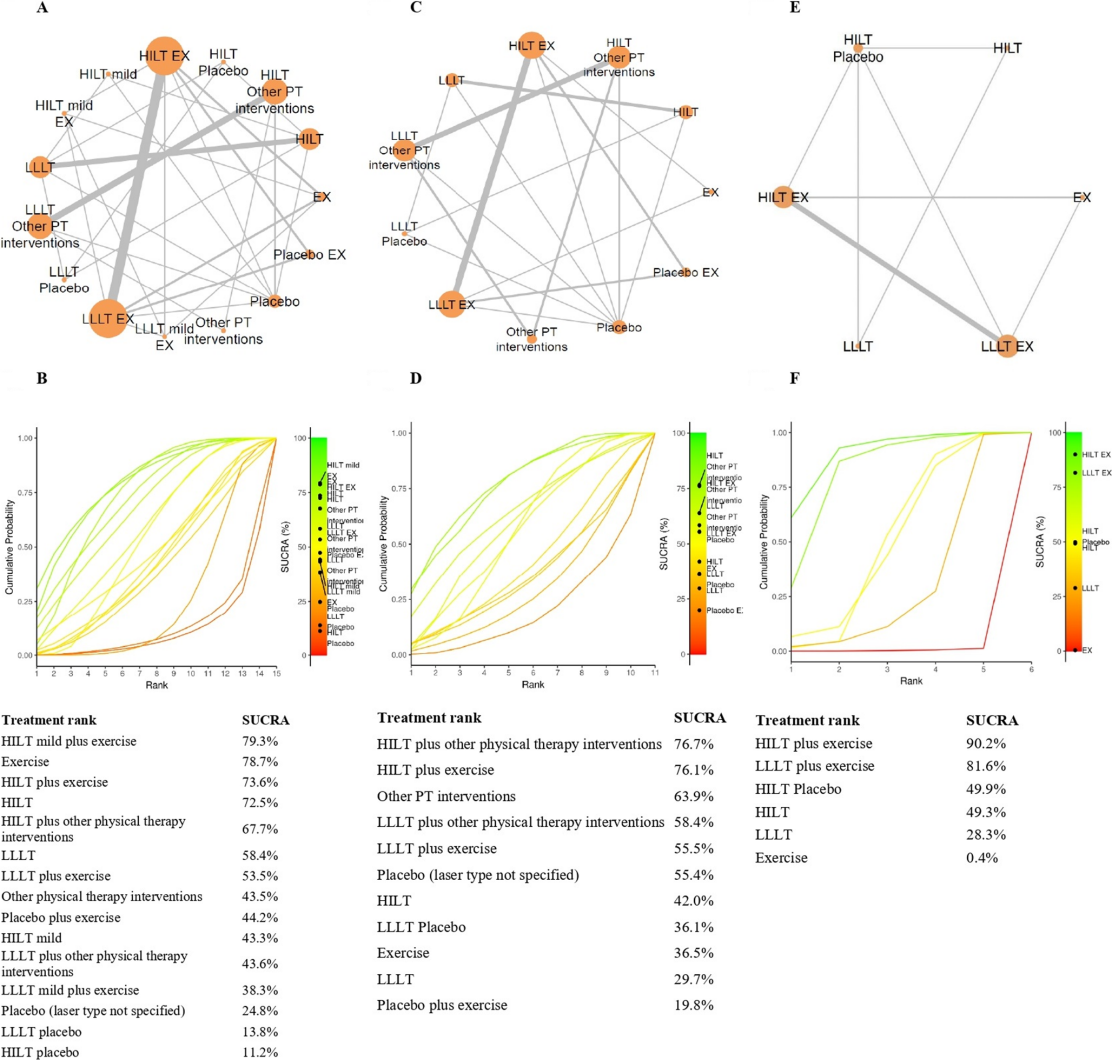
Network plots for pain intensity (Fig. 7A), disability (Fig. 7C), and range of motion (Fig. 7E) after treatment, along with their corresponding surface under the cumulative ranking curve (SUCRA) values (Fig. 7B, Fig. 7D, and Fig. 7 F, respectively), illustrate the comparative effectiveness across laser therapy modalities, either applied alone or in combination with adjunct physiotherapeutic interventions or placebo.

 For the network meta-analysis of ROM outcomes, heterogeneous measurement methods were used across studies (Schober test [38, 42] and goniometry [50–52, 56]). Therefore, all values were normalized to a standardized 0–100 scale to ensure comparability and allow their inclusion in the network meta-analysis. The analysis (Fig. 7E) included six RCTs (three two-arm and three multi-arm trials), with an average change in ROM (pre- and post-treatment) of 12.2% across studies [35, 39, 47–49]. The model showed a good fit (D̄ = 14.7; observed = 15), confirming network connectivity and indicating no evidence of inconsistency (p > 0.05), although moderate heterogeneity was observed (τ ≥ 50%) [29, 30].

 A total of 336 participants contributed to the network, yielding eight direct and 15 potential indirect comparisons. The main contrasts, reflected by larger node sizes and thicker connecting edges, involved HILT versus LLLT, both combined with exercise. The Litmus-O-gram rank plot for ROM outcomes (Fig. 7F) [29], considering the limited number of studies assessing ROM, suggested that HILT combined with exercise (SUCRA = 90.0%) and LLLT combined with exercise (SUCRA = 81.6%) had the highest probabilities of improving ROM, whereas exercise alone showed the lowest probability of effectiveness (SUCRA = 0.4%) [29, 30]. However, exercise alone was evaluated in only a small number of studies [42, 45].

*Change in ROM between baseline and follow-up (4–12 weeks).* No meta-analysis was performed for changes in ROM at follow-up because only two studies reported this outcome—one comparing HILT with LLLT applied alone [38] and one comparing HILT with LLLT combined with exercise [56]. The limited amount of evidence, together with the different combinations of interventions evaluated, made pooling inappropriate, as it would not yield a reliable estimate or allow meaningful assessment of heterogeneity.

### Publication bias

Publication bias was assessed using both visual inspection of funnel plots and statistical testing (Egger’s regression test) for pain intensity and disability outcomes at post-treatment, as these analyses included more than ten RCTs [32], in contrast to ROM outcomes, for which publication bias assessment was not performed due to the limited number of included studies (six RCTs) [38, 42, 50–52, 56]. For pain intensity, evaluated using MDs derived from change scores on the VAS scores [35–38, 42, 44–50, 52, 53, 56], NPRS scores [51], and the KOOS pain subscale [54], visual inspection of the funnel plot did not suggest the presence of publication bias. This finding was supported by Egger’s test, which showed no evidence of funnel plot asymmetry (intercept = 0.54, 95% CI −3.65 to 4.73; t = 0.254; p = 0.802).

**Fig. 8:**
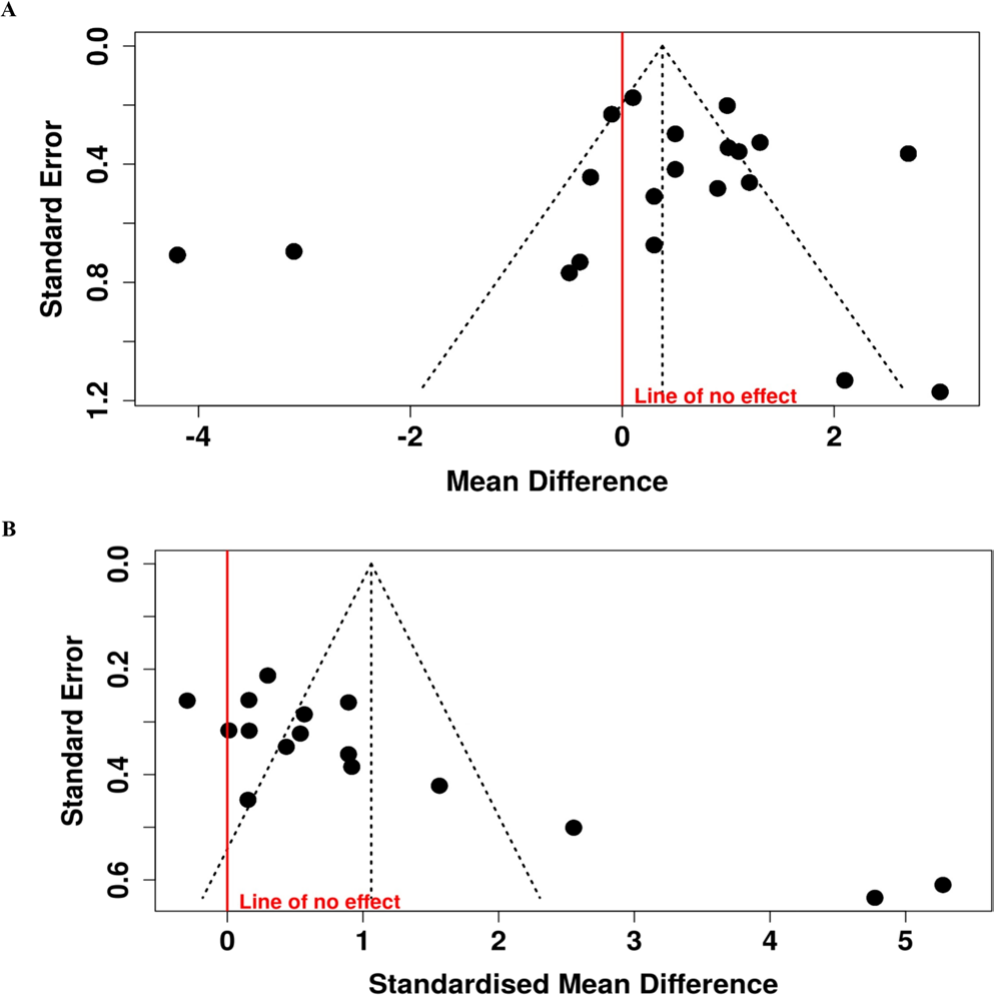
Funnel plots assessing potential publication bias for changes between baseline and post-treatment. Funnel plot for pain intensity, based on MDs derived from change scores in the VAS, NPRS, and KOOS pain subscale (20 studies) (Fig. 8A). Funnel plot for disability, based on SMDs from 16 studies using different instruments, including the Lequesne Index, ODI, NDI, FAOS, BQ-FS, Q-DASH, WOMAC, TUG, and SPADI (Fig. 8B).

 In contrast, disability outcomes—analyzed using SMDs derived from multiple measurement instruments, including the Lequesne Index [35], ODI [38, 42, 49], NDI [37, 50], BQ-FS [44], Q-DASH [47], WOMAC [36, 39, 55, 56], TUG [51], FAOS [40], and SPADI [48, 52]—showed visual asymmetry in the funnel plot, suggesting a potential publication bias (Fig. 8B). This observation was corroborated by Egger’s test, which indicated significant funnel plot asymmetry (intercept = 8.68, 95% CI 5.09 to 12.28; t = 4.734; p < 0.05) [31].

## Discussion

 This SR compared the effectiveness of HILT and LLLT, applied alone or in combination with other physical therapy interventions, for the management of MSDs. Overall, HILT was associated with statistically greater reductions in pain intensity than LLLT, both when applied as a stand-alone intervention and when integrated into exercise-based or multimodal rehabilitation programs. However, these statistically significant differences were not consistently clinically meaningful, as the observed MDs frequently failed to exceed established MCID thresholds for VAS. Findings from the network meta-analysis further suggested a relative advantage of HILT for pain reduction and indicated potential benefits for disability and ROM, particularly when combined with exercise or multimodal physiotherapy approaches. Nevertheless, these findings should be interpreted with caution, as the limited number of available studies, variability in energy dosimetry between HILT and LLLT protocols, and methodological heterogeneity across trials are likely to have influenced the overall strength and precision of the evidence.

### Comparing analgesia for HILT vs. LLLT

 Current evidence supports the analgesic effects of both HILT and LLLT, as reported in multiple SRs and clinical trials [10, 11, 13, 14]. The present review is consistent with these findings, showing that HILT was associated with statistically greater reductions in pain intensity compared with LLLT across various MSDs, primarily assessed using the VAS, NPRS, and the KOOS pain subscale [35–42, 44–53, 56]. In this analysis, pain outcomes were expressed as changes from baseline to post-treatment or follow-up, rather than as single time-point measures, to better capture the magnitude of the therapeutic response and reduce the influence of interindividual variability in baseline pain levels. This methodological approach is consistent with recommendations from the Cochrane Handbook for Systematic Reviews of Interventions, which indicates that change-from-baseline data may provide a more accurate estimate of treatment effects and help minimize bias related to baseline imbalances [25, 58].

 HILT appeared to be particularly effective when applied as a stand-alone intervention (MD = 0.8), especially in conditions such as plantar fasciitis, knee osteoarthritis, and lateral epicondylalgia. Moreover, when HILT was combined with other physical therapy modalities (MD = 1.4), greater analgesic effects were observed in chronic neck pain [37], low back pain [42], lumbar disc herniation [38, 49, 50], carpal tunnel syndrome [43–45], and knee osteoarthritis [35, 36, 39, 51, 53–55]. These statistically significant differences were also observed at follow-up assessments for both laser-only applications (MD = 1.2) [36] and multimodal interventions (MD = 1.1) [39, 44, 46]. At the condition-specific level, the largest MDs favoring HILT were reported in knee osteoarthritis (MD = 1.0), carpal tunnel syndrome (MD = 0.9), lateral epicondylalgia (MD = 0.6), and shoulder disorders (MD = 2.0).

 However, these statistically significant improvements did not consistently translate into clinically meaningful benefits. When contrasted with condition-specific MCIDs for pain assessed using the VAS—generally estimated to range between 1.5 and 2.0 points (95% CI: 1.2–2.0) across different MSDs [33, 58–60]—most of the observed mean differences were below these thresholds and were accompanied by wide confidence intervals, reflecting substantial clinical variability. Accordingly, although HILT demonstrated statistically superior analgesic effects compared with LLLT, these improvements did not consistently exceed established MCID values across conditions. This finding suggests that, while the analgesic efficacy of HILT is supported by quantitative evidence, its clinical relevance may be limited in some contexts, highlighting the importance of interpreting statistical superiority alongside patient-perceived improvement and functional recovery.

 Both HILT and LLLT share similar analgesic mechanisms related to photobiomodulation [6–8]. These mechanisms include reductions in nociceptive nerve conduction velocity, increased release of β-endorphins, and modulation of inflammatory mediators through improvements in local microcirculation [7, 8, 10, 11]. The apparently greater analgesic effects observed with HILT may be partly explained by its additional thermal properties, which can further enhance regional blood flow, facilitate the clearance of nociceptive substances, promote muscle relaxation, and reduce muscle spasm [12, 61, 62].

 This combined photothermal and photobiomodulatory mechanism may be particularly relevant, as several of the included studies targeted peri-lesional regions to influence both local tissues and adjacent musculature, potentially contributing to disruption of the pain–spasm cycle and enhancement of overall therapeutic effects [58, 62]. Thermal agents are commonly incorporated into the management of chronic conditions such as cervical and lumbar pain, disc degeneration, and knee OA—the most frequently investigated condition in this review. In this context, HILT aligns with current clinical practice by providing both non-thermal (photobiomodulatory) and thermal effects [11, 62, 63], a feature that differentiates it from LLLT. Notably, many of the included RCTs applied HILT using a two-phase protocol, consisting of a scanning mode to elicit thermal effects followed by a point mode aimed at analgesia, across a range of conditions including plantar fasciitis [40], chronic neck pain [37], frozen shoulder or impingement syndrome [48, 52], carpal tunnel syndrome [45], lateral epicondylalgia [47], and knee OA [53, 54].

 Another plausible explanation for the apparent advantages of HILT is its capacity to deliver higher total energy within shorter treatment durations as a result of its greater power output [63, 64]. Given the higher cumulative energy administered, it is conceivable that HILT may reach therapeutic thresholds with fewer treatment sessions compared with LLLT. However, this hypothesis remains speculative, as none of the included trials incorporated intermediate assessments that would allow direct comparison of the temporal progression of treatment effects between laser modalities. In addition, HILT protocols generally delivered substantially higher total energy doses than those applied in LLLT, potentially placing the latter at a relative disadvantage. Because LLLT devices operate at lower output power, achieving comparable energy delivery with a single probe often requires prolonged exposure times, which may be impractical for large or multi-point treatment areas. In this context, cluster-type LLLT devices may represent a more efficient alternative, as previously proposed in protocols aimed at enhancing muscle performance [65, 66]. Future trials should aim to standardize energy dosing across modalities to enable more equitable comparisons between HILT and LLLT. Based on the energy ranges reported across included studies, a minimum HILT dose of approximately 300 J per session may represent a reasonable reference threshold for achieving therapeutic effects [35–42, 44–48, 50, 52], typically within treatment programs consisting of six to eight sessions.

 HILT also appeared to demonstrate greater effectiveness when combined with other physical therapy interventions that may potentiate its analgesic effects. For instance, TENS may enhance endogenous opioid release [67]; cryotherapy can reduce nerve conduction velocity [68]; manual therapy and therapeutic ultrasound may alleviate pain and stiffness [69, 70]; and stretching interventions improve muscle flexibility [71]. Such multimodal approaches are widely recommended for the management of chronic MSDs [72, 73]. Nevertheless, most trials included in this review implemented relatively short intervention periods (2–4 weeks). Extending treatment duration to the commonly recommended eight-week timeframe [74] may result in more sustained clinical benefits, as suggested by studies reporting improved outcomes following longer intervention periods.

 Finally, efficacy rankings derived from the network meta-analysis indicated that non-laser physiotherapeutic interventions generally outperformed laser-based treatments for the management of MSDs. Among laser modalities, HILT combined with exercise and HILT integrated with other physical therapy interventions ranked highest, whereas LLLT delivered as an adjunct to another treatment remained more effective than exercise or placebo alone. Although current evidence appears to favor HILT in certain contexts, clinical decision-making should also consider factors such as treatment availability, cost, and accessibility. Notably, the most favorable outcomes for HILT were reported in chronic neck pain [37], knee OA [35], lumbar disc herniation [38], and non-specific chronic pain [42].

### Comparing disability for HILT vs. LLLT

 Assessing disability in clinical trials involving MSDs is of particular importance, as it provides critical insight into patients’ functional limitations and their ability to participate in daily and occupational activities [74]. Changes in disability are closely linked to variations in pain intensity, given that pain reduction often facilitates improvements in mobility, physical performance, and engagement in rehabilitation [74, 75]. Although laser therapy is not primarily intended to directly target disability, inclusion of this outcome in clinical trials is valuable for capturing the broader functional impact of therapeutic interventions.

 Evidence from individual studies suggests that both HILT and LLLT may contribute to reductions in disability across various MSDs. However, when data are pooled in meta-analyses, statistically significant differences favoring HILT tend to emerge, particularly when combined with exercise—where larger effect sizes are observed—or when applied as a stand-alone intervention [35–38, 40, 41, 47, 50]. Nevertheless, the substantial heterogeneity in the instruments used to assess disability limits the interpretability of these findings. Consequently, the observed effects cannot yet be contrasted against condition-specific MCID values, and conclusions are currently restricted to the presence of moderate-to-large effect sizes rather than confirmed clinically meaningful improvements.

 From a probabilistic perspective derived from the network meta-analysis ranking, the combination of HILT with exercise demonstrated the most favorable profile for disability reduction. This observation is consistent with the well-established therapeutic role of exercise, which addresses physical impairments by improving joint mobility, muscle strength, and neuromuscular control, while also promoting behavioral adaptations essential for functional recovery [75, 76]. When used as an adjunct, HILT may further support these effects by reducing pain and inflammation, thereby facilitating greater patient participation and adherence to exercise-based programs. Although LLLT combined with exercise also yielded beneficial outcomes, its overall effectiveness appeared lower than that observed for HILT plus exercise. This difference should be interpreted cautiously, as heterogeneity in LLLT energy dosing—previously noted in relation to analgesic outcomes—may likewise influence disability-related results.

### Comparing ROM for HILT vs. LLLT

 ROM is a key therapeutic target in physical rehabilitation, as it directly influences functional capacity and overall recovery [77]. Both laser modalities have been proposed to enhance ROM through physiological mechanisms such as increased tissue extensibility and reduced stiffness mediated by thermal effects—particularly with HILT [61, 64, 65]. However, analgesia remains the most plausible underlying mechanism for both interventions [11], as pain relief may indirectly improve mobility by reducing muscle spasms and increasing tolerance to movement [62].

 In the network meta-analysis, laser-based interventions consistently ranked higher for ROM improvement when combined with exercise [29]. In contrast, isolated laser applications were generally outperformed by active exercise interventions, which is expected given that exercise directly promotes joint movement, particularly in conditions such as osteoarthritis and low back pain. These findings suggest that laser therapy should be considered an adjunct rather than a stand-alone intervention for improving ROM. Furthermore, the limited number of available studies and the heterogeneity of assessment methods (e.g., goniometry, Schober test) limit the robustness of the evidence and underscore the need for future trials specifically designed to evaluate both the isolated and combined effects of laser therapy on joint mobility*.*

### Differences in penetration depth between HILT and LLLT

 Contrary to the traditional assumption that higher laser power guarantees deeper tissue penetration [10, 12, 14, 78], recent vivo evidence indicates a more complex relationship between power output, wavelength characteristics, and tissue transmission [78]. Comparative analyses of photobiomodulation devices applied to human tendons have shown that low-power, multi-wavelength systems can achieve greater light transmission to deeper tissues while inducing minimal surface heating. In contrast, HILT tends to dissipate a larger proportion of its energy superficially, generating marked thermal effects that may reduce photon availability in deeper layers [78].

 These findings challenge the long-standing notion that HILT inherently provides superior penetration compared with LLLT. Instead, penetration efficiency appears to be primarily influenced by wavelength modulation, pulse duration, and beam dispersion rather than by total power output [76, 79]. Consequently, the clinical advantages of HILT in MSDs may not derive from increased tissue depth of action but from the synergistic interaction between photothermal and photochemical mechanisms—enhancing circulation, modulating nociceptive pathways, and promoting local recovery without necessarily increasing photon diffusion into deeper targets [79].

### Cost-Effectiveness of HILT vs. LLLT

 The cost-effectiveness of HILT compared to LLLT remains a crucial consideration in clinical decision-making. In this SR, HILT demonstrated superior outcomes in pain relief and disability reduction post-treatment, with large and moderate effect sizes, respectively, whether applied alone or in conjunction with exercise or other interventions. However, the limited availability of follow-up studies restricts our ability to determine whether the benefits of HILT endure over time or eventually align with those of LLLT. A significant factor distinguishing these two modalities is their cost (Table 2): HILT devices average 2,700), based on manufacturer reference values. This two- to three-fold price difference raises important questions about whether HILT’s enhanced clinical outcomes are solely attributable to its higher cost. As previously discussed, discrepancies in the total energy delivered during treatment may be pivotal in driving therapeutic effects. If energy dose is indeed the primary determinant of efficacy, it stands to reason that LLLT, when optimized to deliver comparable energy levels, could yield similar results at a significantly lower cost. Moving forward, studies should focus on standardizing and transparently reporting energy dosages to provide clearer insights into the comparative value and clinical applicability of each modality, particularly in resource-constrained environments where cost-effectiveness is essential.

### Alignment with WALT Recommendations

 The WALT provides condition-specific dosage recommendations for LLLT in MSDs [57]. After grouping the included trials by clinical condition, only seven studies were found to adhere to the WALT dosage recommendations [37, 38, 41, 42, 47, 49, 50].

*Knee osteoarthritis.* The treatment parameters reported across the included trials showed no adherence to the WALT recommendations for knee osteoarthritis (780–830 nm, 3–6 points, ≥ 4 J per point, minimum total dose of 12 J, and 20–300 s per point) [57]. Wavelengths and treatment areas were generally consistent with these guidelines; however, substantial variability was observed in energy density (10–50 J/cm²), total energy (8–1,250 J), and treatment duration (2–33 min) [32, 33, 36, 48, 50, 53]. Only one trial that used a 905 nm pulsed laser (0.08 W, 1.5 J/cm², 9 J, six points) [55] closely matched WALT’s protocol for anteromedial knee pain (904 nm, 4–6 points, ≥ 1 J per point, 30–600 s).

*Lumbar spine.* Four studies [38, 42, 49, 50] applied parameters within the WALT-recommended wavelength range for lumbar spine disorders (780–860 nm; 4–8 points; ≥ 4 J per point; minimum total dose of 16 J). Despite meeting these wavelength criteria, considerable variability was observed in power output (0.07–0.64 W), total energy delivered (240–1,200 J), and treatment duration (8–30 min). One trial presented a minor deviation by applying a slightly higher number of irradiation points, whereas another trial employing a 904 nm pulsed laser (0.05 W, 1.8 J/cm², 12 J total, four points, 4 min) demonstrated close adherence to WALT guidance for lumbar spine applications (≈ 1 J per point, 30–600 s) [57].

*Carpal tunnel syndrome.* Neither of the two trials adhered to WALT recommendations, although some individual parameters showed partial alignment [44, 45]. One study employed 860 nm (0.05 W, 8–20 J/cm², ten points, 32–66 s), which coincided with the recommended wavelength and dose but exceeded the advised number of irradiation points (2–3), resulting in non-adherence [45]. The other used a 905 nm pulsed laser (0.025 W, 6 J/cm²); however, the incomplete reporting of key treatment parameters prevented verification of compliance, and it was therefore classified as non-adherent [44].

*Lateral epicondylitis.* The studies followed the WALT recommendations (780–860 nm or 904 nm, 2–3 points, 2–4 J per point, ≤ 100 mW/cm²) [41, 47, 57]. One trial used 808 nm (0.05 W, 8 J/cm², four points) and another used 904 nm (0.16 W, 2.4 J/cm², six points, 216 J) [46]. Both protocols also met the minimum recommended number of irradiation points for LLLT—2 to 3 points for 904 nm and at least 4 points for wavelengths between 780 and 860 nm—and adhered to the advised parameters for energy delivery [57].

*Plantar fasciitis.* The trials addressing plantar fasciitis did not adhere to the WALT recommendations. The 784 nm protocol (3 J/cm², 139 J, scanning over 69 cm²) [46] and the 904 nm protocol (8.4 J/cm², 640 J, three points) [40] represented markedly divergent applications relative to WALT guidelines (≥ 4 J per point and ~8 J total for 780–860 nm; ≥ 2 J per point and ~4 J total for 904 nm) [57]. The former employed a scanning technique rather than punctual irradiation, resulting in substantially lower energy delivered per point, whereas the latter delivered an excessively high total energy despite meeting the WALT-recommended minimum number of irradiation points for 904 nm lasers.

*Supraspinatus tendinopathy.* Only one study comparing laser therapies in patients with subacromial impingement syndrome used a mixed-wavelength device (810/980 nm; 0.2 W; 20 J/cm²; 200 J total; ten points, 20 J per point) [48], and this protocol did not adhere to WALT recommendations. It exceeded the recommended wavelength ranges for therapeutic LLLT—780–860 nm for continuous-wave applications and 904 nm for pulsed lasers—as well as the dosing parameters associated with each spectrum (≥ 4 J per point and ~8 J total for 780–860 nm; ≥ 2 J per point and ~4 J total for 904 nm). The protocol also surpassed the advised number of irradiation points (2–3 points), further demonstrating limited adherence to established WALT dosing guidelines [57].

*Cervical spine.* The single included study (830 nm, 0.1 W, 4 J/cm², 300 J total, eight points) demonstrated strong adherence to WALT guidelines for this wavelength range (780–860 nm, 4–12 points, ≥ 4 J per point, ≥ 16 J total) [36, 57], delivering energy within an appropriate therapeutic window.

 Overall, adherence to WALT recommendations appeared to influence the consistency and variability of treatment outcomes more than the absolute magnitude of effect in comparisons between HILT and LLLT. When only WALT-compliant protocols were considered, the differences between both laser modalities were attenuated and did not reach the threshold for clinical relevance (<10% change) [32]. This pattern suggests that appropriate dosimetry—encompassing total energy, energy density, and the number of irradiation points or treatment area—rather than device power alone, may be the key determinant of therapeutic response. In contrast, the larger effects observed in non-adherent protocols were accompanied by substantial heterogeneity and very low certainty of evidence, indicating that inconsistent or suboptimal dosing may overestimate the apparent benefits of HILT. Collectively, these findings underscore the need for future clinical trials to align with WALT dosing parameters and contemporary photobiomodulation guidelines to enhance methodological comparability [57, 80, 81], optimize treatment outcomes, and strengthen the evidence base for laser therapy in musculoskeletal disorders.

### Limitations

 This review has several limitations that should be acknowledged. Considerable heterogeneity was observed across several meta-analyses, likely driven by differences in sample sizes, study designs, intervention protocols, and laser parameters. This heterogeneity could not be reduced by excluding studies with a high RoB, as the limited number of trials available for pairwise comparisons restricted the feasibility of conducting sensitivity analyses. Together with concerns related to indirect evidence—arising from the combination of different clinical conditions and variable dosimetric approaches—these factors substantially contributed to the very low certainty ratings assigned using the GRADE framework.

 Another limitation concerns the diversity of instruments used to assess key outcomes, which necessitated the use of SMDs, particularly for disability and ROM. Although SMDs allow the pooling of data derived from different measurement tools, they inherently reduce clinical interpretability and complicate the determination of whether observed effects exceed the MCID for the instruments used. While HILT demonstrated statistically superior analgesic effects—and in some comparisons, disability improvements—relative to LLLT, it remains uncertain whether these differences translate into clinically meaningful benefits. To enhance comparability and clinical applicability, future RCTs should incorporate standardized, validated, and condition-specific outcome measures, ideally enabling the calculation of MDs and a clearer assessment of clinical significance.

 A further limitation relates to the substantial variability in laser parameters, particularly regarding HILT dosimetry, which challenges the definition of a clear therapeutic window. The absence of standardized protocols highlights the need to develop evidence-based dosing guidelines to optimize therapeutic effectiveness and promote consistency across studies. In the case of LLLT, deviations from WALT-recommended parameters—especially concerning total energy for musculoskeletal conditions, power density, fluency, and treatment area—further emphasize the importance of adhering to standardized dosimetric criteria. Such variability may have influenced treatment outcomes, as deviations from the therapeutic window can alter the photobiomodulatory response and reduce clinical efficacy.

### Clinical implications

From a clinical standpoint, these findings suggest that although HILT provides statistically superior analgesic effects compared with LLLT in some comparisons, the magnitude of improvement may not justify the routine replacement of LLLT in standard rehabilitation settings. Rather, HILT may represent a potential option in selected patients, such as those with chronic or refractory musculoskeletal pain, where higher energy delivery could contribute to symptom relief when integrated into multimodal physiotherapy programs.

## Conclusion

This SR evaluated the comparative effects of HILT and LLLT on pain, disability, and ROM in musculoskeletal conditions. Across several comparisons, HILT demonstrated statistically greater pain reduction than LLLT, particularly when combined with exercise or multimodal physical therapy approaches. However, it is important to emphasize that statistical significance does not necessarily translate into clinically meaningful benefit. In multiple comparisons, the observed pain reductions did not exceed established MCID thresholds, suggesting that the magnitude of improvement may be of limited relevance in clinical practice. Regarding disability outcomes, a potential advantage was observed only for HILT combined with exercise, with effect sizes of moderate magnitude. Nevertheless, the considerable heterogeneity of the instruments used to assess disability limits the interpretability and clinical extrapolation of these findings, precluding firm clinical conclusions. Similarly, ROM outcomes were reported in a limited number of studies and assessed using heterogeneous methodologies, which restricted comparability and resulted in no consistent or clinically meaningful differences between modalities.

 Differences in total delivered energy dose may partly explain the observed effects, as HILT protocols typically apply substantially higher total energy than those used in LLLT. Despite this, the current body of evidence remains insufficient to establish the clinical superiority of one modality over the other, as most effect estimates are based on very low-certainty evidence and are therefore highly uncertain and likely to change with future high-quality RCTs.

 Notably, studies evaluating LLLT that reported closer adherence to the WALT recommendations tended to demonstrate more favorable outcomes, underscoring the potential influence of dosimetric rigor on treatment effects. Future studies comparing both laser modalities should align LLLT protocols with the posological guidelines proposed by WALT and develop specific dosimetric recommendations for HILT to improve methodological consistency and clinical applicability. Additionally, longer-term follow-up assessments are warranted, as most included studies were limited to end-of-treatment evaluations.

 In conclusion, based on the current evidence, both HILT and LLLT may be considered therapeutic options for pain management in musculoskeletal conditions. However, their relative clinical benefits do not appear to differ clearly, and treatment decisions should be made cautiously within a multimodal rehabilitation framework, considering patient characteristics, dosimetric parameters, and the low certainty of the available evidence.

## Supplementary Information

Below is the link to the electronic supplementary material.

Supplementary Material 1 (DOCX 33.0 KB)

## Data Availability

The datasets used and/or analysed during the current study are available from the corresponding author on reasonable request.

## Abbreviations

CI: Confidence intervals
CG: Control group
DASH: Disabilities of the Arm, Shoulder, and Hand
DIC: Deviance Information Criterion
EG: Experimental group
GRADE: The Grading of Recommendations, Assessment, Development, and Evaluation
HILT: High-intensity laser therapy
KOOS: Knee Injury and Osteoarthritis Outcome Score
LLLT: Low-level laser therapy
Dbar: Mean Deviance
MSDs: Musculoskeletal disorders
NPRS: Numeric Pain Rating Scale
ODI: Oswestry Disability Index
PEDro: The Physiotherapy Evidence Database
PPT: Pressure pain threshold
PRISMA: Preferred Reporting Items for Systematic Reviews and Meta-Analyses
RCTs: Randomized controlled trials
ROB2: Cochrane Risk of Bias 2 tool
RMQ: the Roland-Morris Questionnaire
SPADI: Shoulder Pain and Disability Index
SMD: Standardized mean differences
SR: Systematic review
SUCRA: Surface under the cumulative ranking curve
TENS: Transcutaneous electrical nerve stimulation
VAS: Visual Analog Scale
WOMAC: Western Ontario and McMaster Universities Osteoarthritis Index
