# Supplementary material for: Comparison of the effectiveness of high-intensity laser therapy versus low-level laser therapy in musculoskeletal disorders: a systematic review and network meta-analysis
Source: Lasers Med Sci. 2026 Feb 16;41(1):30. doi: 10.1007/s10103-026-04812-9 (PMC12909518; doi:10.1007/s10103-026-04812-9)
Supplement: Supplementary file 1 — Supplementary Material 1 (DOCX 33.0 KB) [file 10103_2026_4812_MOESM1_ESM.docx]

**Appendix 1**. Search strategy (last updated December 13, 2025).

| Search | Keywords | Identification of studies via databases and registers | | | | | | | | Identification of studies via other methods |
| --- | --- | --- | --- | --- | --- | --- | --- | --- | --- | --- |
|  |  | PubMed | Scopus | Web of Science | EBSCOhost | ScienceDirect | Cochrane Library | PEDro | **Total** | Google Scholar |
| S1 | *"Lasers"* | 102,064 | 1,760,896 | 1,298,933 | 430,548 | 1,000,000 | 27,203 |  | 4,619,644 |  |
| S2 | *"Laser Therapy"* | 52,165 | 50,677 | 16,277 | 137,778 | 24,115 | 8,052 |  | 289,064 |  |
| S3 | *"Low-Level Light Therapy"* | 8,957 | 5,596 | 869 | 20,637 | 483 | 1,918 |  | 38,460 |  |
| S4 | *"High-Intensity Laser Therapy"* | 206 | 292 | 322 | 677 | 132 | 245 |  | 1,874 |  |
| S5 | *"Class IV laser"* | 33 | 63 | 143 | 108 | 169 | 35 |  | 551 |  |
| S6 | *S1 OR S2 OR S3 OR S4 OR S5 OR S6* | 140,467 | 1,761,050 | 191,741 | 527,271 | 1,024,899 | 27,443 |  | 3,672,871 |  |
| S7 | *"Musculoskeletal Pain"* | 13,253 | 22,604 | 15,866 | 39,967 | 25,727 | 3,434 |  | 12,0851 |  |
| S8 | *"Musculoskeletal diseases"* | 20,357 | 54,091 | 4,000 | 75,508 | 14,931 | 5,930 |  | 174,817 |  |
| S9 | *"Neck Pain"* | 18,964 | 37,332 | 17,135 | 60,488 | 33,160 | 6,147 |  | 173,226 |  |
| S10 | *"Myofascial Pain Syndromes"* | 2,347 | 3,452 | 448 | 8,219 | 3,170 | 1,908 |  | 19,544 |  |
| S11 | *"Low Back Pain"* | 49,176 | 88,264 | 63,039 | 156,662 | 70,873 | 15,707 |  | 443,721 |  |
| S12 | *"Osteoarthritis"* | 134,331 | 189,226 | 143,243 | 387,973 | 181,458 | 27,317 |  | 1,063,548 |  |
| S13 | *"Pain Management"* | 93,902 | 75,240 | 47,167 | 341,880 | 96,458 | 18,661 |  | 673,308 |  |
| S14 | *S7 OR S8 OR S9 OR S10 OR S11 OR S12 OR S13* | 314,142 | 434,871 | 270,996 | 1,007,566 | 362,674 | 72,696 |  | 2,462,945 |  |
| S15 | S7 AND S14 | 1,345* | 3,686* | 945* | 4,725* | 2.004* | 1,166* | 11* | 13,882* | 7,060** |

*Search algorithm used for formal databases: *("Lasers" OR "Laser Therapy" OR "Low-Level Light Therapy" OR "High-Intensity Laser Therapy" OR "Class IV laser") AND ("Musculoskeletal Pain" OR "Musculoskeletal diseases" OR "Neck Pain" OR "Myofascial Pain Syndromes" OR "Low Back Pain" OR "Osteoarthritis" OR "Pain Management")*

***Search algorithm used for alternative methods or registers: ("Laser Therapy" OR "Low-Level Light Therapy" OR "High-Intensity Laser Therapy") AND ("Musculoskeletal Pain" OR "Musculoskeletal diseases")*

**Appendix 2**. Summary of excluded articles via databases, registries, and other methods.

| N° | Source | Reason of exclusion | Author (year) | Reference |
| --- | --- | --- | --- | --- |
| 1 | Studies via other methods | Osteoporosis | Thabet et al. (2011) | Thabet AA, Mohamed MS, Ibrahim MM, Helal OF. High intensity laser versus low intensity laser therapy in management of postmenopausal osteoporosis. Bull Faculty Phys Therapy. 2011;16(1):73. |
| 2 | Studies via database and registers | Bell´s palsy | Alayat et al. (2014) | Alayat MSM, Elsodany AM, El Fiky AAR. Efficacy of high and low level laser therapy in the treatment of Bell’s palsy: a randomized double blind placebo-controlled trial. Lasers Med Sci. 2014;29(1):335–42. http://dx.doi.org/10.1007/s10103-013-1352-z |
| 3 | Studies via database and registers | Healthy subjects | Zwolińska et al. (2014) | Zwolińska J, Weres A, Kwolek A, Furgał W. Assessment of the effectiveness of low-level laser therapy (LLLT) – and high intensity laser therapy (HILT) to reduce the symptoms of muscle fatigue – a comparative analysis. Polish Journal of Sports Medicine 2014; 30(3). |
| 4 | Studies via database and registers | Not considered an outcome of interest for this review. | Kulchitskaya et al. (2017) | Kulchitskaya DB, Konchugova TV, Fedorova NE. Comparative evaluation of the effects of high-intensity and low-intensity laser radiation on microcirculation among patients with knee arthritis. J Phys Conf Ser. 2017;826:012015. http://dx.doi.org/10.1088/1742-6596/826/1/012015 |
| 5 | Studies via database and registers | Dysmenorrhea | Thabet et al. (2021) | Thabet AA, Ebid AA, El-Boshy ME, Almuwallad AO, Hudaimoor EA, Alsaeedi FE, et al. Pulsed high-intensity laser therapy versus low level laser therapy in the management of primary dysmenorrhea. J Phys Ther Sci. 2021;33(9):695–9. http://dx.doi.org/10.1589/jpts.33.695 |
| 6 | Studies via database and registers | Not considered an outcome of interest for this review | Taradaj et al. (2019) | Taradaj J, Rajfur K, Rajfur J, Ptaszkowski K, Ptaszkowska L, Sopel M, et al. Effect of laser treatment on postural control parameters in patients with chronic nonspecific low back pain: a randomized placebo-controlled trial. Braz J Med Biol Res. 2019;52(12):e8474. http://dx.doi.org/10.1590/1414-431X20198474 |
| 7 | Studies via database and registers | Systematic review | Ahmad et al. (2022) | Ahmad MA, A Hamid MS, Yusof A. Effects of low-level and high-intensity laser therapy as adjunctive to rehabilitation exercise on pain, stiffness and function in knee osteoarthritis: a systematic review and meta-analysis. Physiotherapy. 2022;114:85–95. http://dx.doi.org/10.1016/j.physio.2021.03.011 |
| 8 | Studies via other methods | Not considered an outcome of interest for this review. | Wibisono et al. (2024) | Wibisono K, Handoyo R, Setiawati E. Comparison of the effectiveness of high intensity laser therapy (HILT) and low-level laser therapy (LLLT) on improving balance in knee osteoarthritis. Medica Hospitalia J Clin Med. 2024;11(1):33–7. http://dx.doi.org/10.36408/mhjcm.v11i1.984 |

**Appendix 3**. Assessment of methodological quality of included studies using the PEDro scale.

| **Author (year) Country** | **PEDro scale criteria** | | | | | | | | | | |  |
| --- | --- | --- | --- | --- | --- | --- | --- | --- | --- | --- | --- | --- |
|  | **CRITERIA**  **1*** | **CRITERIA 2** | **CRITERIA**  **3** | **CRITERIA**  **4** | **CRITERIA**  **5** | **CRITERIA**  **6** | **CRITERIA**  **7** | **CRITERIA**  **8** | **CRITERIA**  **9** | **CRITERIA**  **10** | **CRITERIA**  **11** | **SCORE** |
| Gworys (2012)^++^ [31] Poland | Yes | Yes | No | Yes | No | No | No | Yes | No | Yes | Yes | 5/10 |
| Kheshie (2014)^+^ [32] Saudi Arabia | Yes | Yes | No | Yes | Yes | No | Yes | Yes | No | Yes | Yes | 7/10 |
| Alayat (2017)^+^ [33] Egypt | No | Yes | No | Yes | Yes | Yes | Yes | No | No | Yes | Yes | 7/10 |
| Taradaj (2018)^++^ [34] Poland | Yes | No | Yes | Yes | Yes | No | Yes | Yes | Yes | Yes | Yes | 8/10 |
| Dekholsh (2018)^++^ [35] Iran | Yes | Yes | No | Yes | Yes | No | No | No | No | Yes | Yes | 5/10 |
| Ordahan (2018)^+^ [36] Turkey | Yes | Yes | Yes | Yes | No | Yes | Yes | Yes | No | Yes | Yes | 8/10 |
| Fekri (2019)^++^ [37] Iran | Yes | Yes | No | Yes | No | No | No | Yes | No | Yes | Yes | 5/10 |
| Abdelbasset (2020)^+^ [38] Saudi Arabia | Yes | Yes | No | Yes | No | No | Yes | Yes | Yes | Yes | Yes | 7/10 |
| Sudiyono (2020)^++^ [39] Indonesia | Yes | Yes | No | Yes | No | No | Yes | Yes | No | Yes | Yes | 5/10 |
| Hojjati (2020)^++^ [40] Iran | Yes | Yes | Yes | Yes | Yes | No | No | Yes | No | Yes | Yes | 7/10 |
| Ezzati (2020)^+^ [41] Iran | Yes | Yes | Yes | Yes | Yes | No | Yes | Yes | Yes | Yes | Yes | 9/10 |
| Naruseviciute (2020)^+^ [42] Lithuania | Yes | Yes | Yes | Yes | Yes | No | No | Yes | No | Yes | Yes | 7/10 |
| Kaydok (2020)^+^ [43] Turkey | Yes | Yes | Yes | Yes | Yes | No | Yes | Yes | Yes | Yes | Yes | 9/10 |
| Zaki (2021)^+^ [44] Iran | Yes | Yes | Yes | Yes | No | No | Yes | Yes | Yes | Yes | Yes | 8/10 |
| Al-Kurdı (2022)^++^ [45] Turkey | Yes | Yes | No | Yes | Yes | No | No | No | No | Yes | Yes | 5/10 |
| Abdelsalam (2022)^++^ [46] Egypt | Yes | Yes | Yes | Yes | Yes | No | No | Yes | No | Yes | Yes | 7/10 |
| Ahmad (2023)^+^ [47] Malaysia | Yes | Yes | Yes | Yes | No | Yes | Yes | Yes | Yes | Yes | Yes | 9/10 |
| Ordahan (2023)^+^ [48] Turkey | Yes | Yes | Yes | Yes | No | Yes | Yes | Yes | Yes | Yes | Yes | 9/10 |
| Astri (2023)^++^ [49] Indonesia | Yes | Yes | No | Yes | No | No | No | Yes | No | Yes | Yes | 5/10 |
| Rizky (2024)^++^ [50] Indonesia | Yes | Yes | No | Yes | No | No | No | Yes | No | Yes | Yes | 5/10 |
| Ezzati (2020)^++^ [51] Iran | Yes | Yes | Yes | Yes | Yes | No | No | Yes | No | Yes | Yes | 7/10 |
| Şen (2025)^++^ [52] Turkey | Yes | Yes | Yes | Yes | Yes | Yes | No | Yes | No | Yes | Yes | 8/10 |

**PEDro (Physiotherapy Evidence Database) scale criteria:**

(1) Eligibility criteria were specified; (2) Random allocation; (3) Concealed allocation; (4) Baseline comparability; (5) Blind subjects; (6) Blind therapists; (7) Blind assessors; (8) Adequate follow-up; (9) Intention-to-treat analysis; (10) Between-group comparisons; (11) Point estimates and variability

*Eligibility criteria does not contribute to total score.

⁺A confirmed score in the Pedro database.

++Score determined by researchers (Not available in PEDro database).
